# Automated pipeline for operant behavior phenotyping for high-throughput data management, processing, and visualization

**DOI:** 10.1038/s44277-025-00046-9

**Published:** 2025-10-24

**Authors:** Sunwoo Kim, Yunyi Huang, Uday Singla, Andrew Hu, Sumay Kalra, Alex A. Morgan, Benjamin Sichel, Dyar Othman, Lieselot L. G. Carrette

**Affiliations:** Department of Psychiatry, University of California - San Diego, La Jolla, CA 92093 USA

**Keywords:** Reward, Research management

## Abstract

Operant behavior paradigms are essential in preclinical models of neuropsychiatric disorders, such as substance use disorders, enabling the study of complex behaviors including learning, salience, motivation, and preference. These tasks often involve repeated, time-resolved interactions over extended periods, producing large behavioral datasets with rich temporal structure. To support genome-wide association studies (GWAS), the Preclinical Addiction Research Consortium (PARC) has phenotyped over 3000 rats for oxycodone and cocaine addiction-like behaviors using extended access self-administration, producing over 100,000 data files. To manage, store, and process this data efficiently, we leveraged Dropbox, Microsoft Azure Cloud Services, and other widely available computational tools to develop a robust, automated data processing pipeline. Raw MedPC operant output files are automatically converted into structured Excel files using custom scripts, then integrated with standardized experimental, behavioral, and metadata spreadsheets, all uploaded from Dropbox into a relational SQL database on Azure. The pipeline enables automated quality control, data backups, daily summary reports, and interactive visualizations. This approach has dramatically improved PARC’s high-throughput phenotyping capabilities by reducing human workload and error, while improving data quality, richness, and accessibility. We here share our approach, as these streamlined workflows can deliver benefits to operant studies of any scale, supporting more efficient, transparent, reproducible, and collaborative preclinical research.

## Lay summary

To uncover why some individuals are more vulnerable to addiction, the PARC rat GWAS and Biobank projects are testing thousands of rats, generating hundreds of thousands of behavioral data files. We created an automated system using Dropbox and Microsoft Azure to organize, store, and visualize these data. This pipeline reduces errors, saves time, and improves sharing, enabling large-scale, high-quality behavioral studies in preclinical models.

## Introduction

Operant paradigms play a central role in preclinical models of neuropsychiatric disorders, enabling the study of complex behaviors, including learning, salience, motivation, and preference, through drug self-administration [1–3], opto-intracranial self-stimulation (ICSS) [4], probabilistic reversal learning [5], effort-based decision-making [6], and timeout-based [7] or complex sequence [8] tests. These tasks often involve repeated, time-resolved interactions over extended periods of time, producing large behavioral datasets. Moreover, large numbers of animals can be required to achieve significance, like to evaluate the effect of the genotype in genome-wide association studies (GWAS; N > 1000), which are accumulated over several cohorts. Besides data collection, efficient data management can become a bottleneck. Manual handling of large-scale data is labor-intensive, error-prone, and possibly inconsistent across experimenters, hindering reproducibility and analysis. Moreover, it often requires simplifications that limit the depth and dimensionality of the analysis. The computational revolution presents opportunities to address this challenge. The conceptual prototype Behavflow demonstrated how automated data pipelines can improve efficiency and reduce experimenter workload by providing experiment-level tracking and real-time processing of operant data with summary statistics and visualizations [9]. MouseBytes, an open-access high-throughput pipeline and database specifically for rodent touchscreen-based cognitive assessment [10], moreover, shows the benefits of improved standardization, reproducibility, and collaboration.

**Fig. 1:**
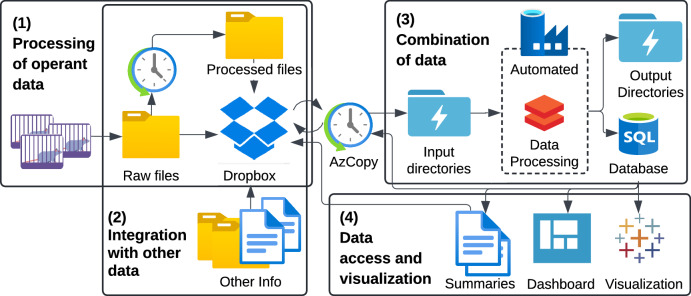
General flowchart of the 4-part automated pipeline. 1) Processing of raw operant TXT files into Excel output files (Fig. 2). 2) Integration of the operant data with other data in standardized files, like Cohort Information, Daily issues, Exits, and other behavioral test data (Fig. 3). 3) Consolidation of all the data for storage in a relational database using Databricks and Data Factory pipelines on Azure connected to Dropbox through AzCopy for file transfer (Fig. 4). 4) Additional data access and visualization through summary files, an online dashboard, and the integration with Tableau (Fig. 5). The different components of the pipeline are discussed in greater detail below.

Here, we present a robust and scalable automated pipeline built to manage, analyze, and share the 100,000 files of operant behavior data generated for the characterization of cocaine [2] and oxycodone [3] addiction-like behavior in over 2,000 animals for GWAS, using a 4-part strategy (Fig. 1). First, raw operant files are automatically processed into standardized Excel output files on Dropbox. Second, other behavioral tests, experimenter notes, and metadata on animals, experiments, and cohorts are also stored in standardized files on Dropbox. Next, all this data are processed and integrated into a relational SQL database on Microsoft Azure. Finally, this database is used to provide data feedback, through the generation of experiment, animal, cohort, and population summaries and visualization of the data.

## Materials and methods

### Animals, drugs, and behavioral characterization

As the behavioral procedures are not central to this paper, we defer to previous publications [1–3] and the supplementary materials and methods, where more extended descriptions and a procedural timeline (Fig. S1) can be found. Briefly, animals are implanted with jugular vein catheterization for intravenous (*i.v*.) drug self-administration through lever pressing in operant chambers (Med Associates). Active lever presses result in drug infusion followed by a 20-second timeout period; inactive lever presses have no consequence. There is short sessions (ShA), long sessions (LgA), sessions with a progressive ratio (PR) of reinforcement and sessions with foot shocks. Each animal was implanted with a unique RFID for identification and tracking. All procedures complied with the NIH guidelines and were approved by the Institutional Animal Care and Use Committees of The Scripps Research Institute and University of California San Diego.

**Input Data** all stored using standardized Excel templates in designated folders on Dropbox:

- **Operant data processing**: GetOperant [11] is scheduled via Microsoft Task Scheduler to automatically convert MedPC session TXT files (Suppl. File 1) into Excel output files (Suppl. File 2) [GitHub: SRC/Preprocessing-Operant-Data]. Each Excel output file represents one session per computer, with rats in columns and extracted variables in rows: total drug infusions, active, and inactive lever presses, timestamps for each event, session start and end time and date, box number, session program, and the raw file name. Additional metrics extracted for specific session types included breakpoint (PR sessions), total number of shocks, and infusions following shock (shock sessions). Session metadata are systematically encoded in the filename to include location (building and room e.g., MTF134), controlling computer (e.g., A-E), cohort number (e.g., C01- C50), drug (e.g., OXY or COC), and session ID (e.g., LGA10, SHA04, PR03).
- **Cohort Information file** (Suppl. File 3): Each row represents a unique animal and includes: subject ID, RFID, sex, cohort designation, experimental group (e.g., drug, saline, sham, naive), drug group (oxycodone or cocaine), any other individual characteristics (e.g., date of birth, coat color, ear marks, parentage), experimental metadata (e.g., dates, technician IDs, assigned treatments, dissection groups), and additional experimental data (e.g., weekly weights, catheter patency test outcomes).
- **Daily Issues file** (Suppl. File 4): Records session and animal-level experimenter observations per cohort. Each row includes: animal ID, RFID, date, session ID, standardized issue code (options: disconnected, tangled, empty syringe, sick, or other), keep/discard decision, decision type (objective/subjective), and optional additional information in notes.
- **Exit file** (Suppl. File 5): Records animals excluded from the study. Each row represents a unique animal and includes: subject ID, RFID, cohort, exit date, last good session, exit code (options: Death, Brevital Fail, Other), decision type (objective/subjective), status of key behavioral testing, status of tissue collection, and additional notes. Animals that died before behavioral testing are replaced as indicated.
- **Behavioral test files**: Records collected measurements and processed data, with each row representing a unique rat. Von Frey (Suppl. File 6) records force and latency measurements per paw, averaged per timepoint and the difference between them. Tail immersion (TI; Suppl. File 7) records latencies for tail withdrawal before ShA, with and without oxycodone on board, after LgA with oxycodone on board, and the difference between timepoints. Bottle brush (IRR; Suppl. File 8) records aggressive and defensive behavior counts per observer and averaged per timepoint and the difference between them (as buttle brush tests are discontinued there is actually only 1 file combining all cohorts)
- **Correction Record file:** A dynamic CSV that logs filenames of modified Excel output files requiring reprocessing in Azure. This file is scanned daily and cleared after successful updates.

### Azure cloud integration, database structuring, and data curation [GitHub: SRC/Combination-in-Relational-Database]

- **Data uploading**: New data on Dropbox are uploaded daily to Azure Data Lake using AzCopy scripts triggered by Microsoft Task Scheduler. Modified files are copied too, when listed on the Corrections Record file [GitHub: ../Automated_copy].
- **Data processing**: Within Azure Databricks, 9 dedicated pipelines process the input data into CSV files combined over cohorts suitable for SQL ingestion, with automatic execution orchestrated by Data Factory [GitHub: ../Automated_processing]:
  - The Excel output files are transposed, processed, and combined in a CSV file per session type (ShA, LgA, PR, and Shock, Fig. S2-Top). Filenames are parsed using Regex to extract session metadata. Each row represents a unique rat-session pairing with summary metrics and timestamp arrays as lists.
  - The Cohort Information files are split into a subject table (RFID, experiment group, drug group, sex, experiment dates, etc.; Fig. S2-Middle) and a measurement table (weights, dates, technician IDs; Fig. S2-Bottom) and combined per type in a CSV file. Moreover, the RFID is extracted as unique identifier in the relational database schema.
  - The other behavioral test files for tail immersion, irritability, and Von Frey tests are combined per test in a single CSV file.
- **Data combination in SQL database:** Daily, after successful processing, all data are ingested and stored into a live, raw SQL database, structured in different tables connected through RFID as primary key (Suppl. List 1). Additionally, all tables are joined in a single combined table, generating the raw combined database (Suppl. List 2). During the combination, records with session issues or exits are excluded [GitHub:../Automated_combination]. At timed intervals, a curated version of the raw combined database, known as the stable database (Suppl. List 3), is generated manually using Databricks [GitHub:../Stable_calculations], performing:
  - Outlier removal: Drug infusions >250 (syringe capacity) trigger removal of infusions and active lever presses, unless when there are multiple outliers (indicating a high pressing animal) resulting in capping the infusions at 250.
  - Missing data imputation: Single-session gaps are interpolated linearly (average); edge cases filled using nearest-neighbor method (average 2 previous or following). Multiple consecutive missing sessions are not imputed.
  - Dependent variable calculation: Summary metrics and addiction-relevant phenotypes calculated (list and formulas see Suppl. List 3).

### Data output [Github: SRC/Data-Output-and-Visualization/]

- **Backup copy of the relational databases**: Using AzCopy and Microsoft Task Scheduler, CSV backups of the raw and stable combined databases are automatically saved to a designated Dropbox folder [GitHub: ../Backup-Database/azuretodropboxcombined.py].
- **Behavior traces:** A PDF report is automatically generated using Matplotlib [12] and updated daily in a designated Dropbox folder (Fig. S3). Each page graphically represents all collected behavior data of an animal, including operant drug infusions, active and inactive lever presses during ShA, LgA, PR, Shock, as well as withdrawal latency from tail immersion or force from Von Frey, against the cohort average with uncertainty interval and subject metadata, including recoded issues and exclusion status [GitHub: ../Behavior-Analysis-Automation-and-Graph-Generation: graph_cocaine.py and oxy_graph generation.py].
- **Behavior file:** A cohort-wide operant behavior summary is automatically created and updated daily in a designated Dropbox folder (Suppl. File 9). The first 4 columns, subject ID, RFID, drug group (cocaine or oxycodone), and experiment group (drug vs naive) are pulled from the Cohort Information file. The last 4 columns, last good session, exit date, code, and notes, are pulled from the Exit file. The header consists of the date of the session, which is used for organizing the data and a row with the session ID, pulled from the Excel output files. The next 2 rows identify session issues, one coded, the other expanded with notes, pulled from the Daily Issues file. Within this matrix the number of drug infusions are listed, as extracted from the Excel output files. The matrix is repeated for active and inactive lever presses (in addition to breakpoint for PR or total shocks and first infusion that got shocked for shock sessions) [GitHub: ../Behavior-Analysis-Automation-and-Graph-Generation: cocaine_behavior_sheet_automation.py and oxy_behavior_sheet_automation.py].
- **Interactive visualization**: A web-based dashboard hosted via pythonanywhere provides real-time visualization of Excel output files. Plotly Dash displays session timelines of events (infusions, lever presses, timeouts) and histograms of inter-infusion intervals with options for zooming, panning, and animal selection [GitHub: ../Interactive-Visualization].
- **Tableau visualization** [13]: A GUI connected to the Azure SQL database provides customizable visual analytics templates. Data points can be color-coded based on metadata, like sex, drug, cohort, or AI, and hovering over data points with the cursor reveals their identity through pop-up information. Tableau Public dashboards are manually updated following stable database releases.

## Results

### High-throughput characterization of addiction-like behaviors for GWAS and the rat biobanks

Extended access self-administration models provide high construct, face, and predictive validity for substance use disorders as they reproduce escalation of intake [14–16], increased motivation [17], continued use despite adverse consequences [18, 19], increased choice over natural rewards [20, 21], withdrawal-related behaviors [22–24], and relapse [25, 26], mirroring key diagnostic features of substance use disorder. For these reasons, this model is used to characterize oxycodone and cocaine addiction-like behaviors in genomically diverse heterogeneous stock rats for GWAS and the associated rat addiction biobanks at the Preclinical Addiction Research Consortium (PARC) [1]. Each animal undergoes a standardized behavioral protocol (see Supplementary Behavioral Procedures and Fig. S1), generating operant and non-operant behavioral data over the course of several weeks. Across thousands of subjects, this produced >100,000 files over several years. Key behavioral endpoints include total drug intake, active/inactive lever pressing, progressive ratio breakpoint, response to punishment, irritability-like behavior, nociception, analgesia, and response to treatment interventions. Animals are characterized based on individual behavioral measures or derived dependent variables, like the composite Addiction Index (AI) [27–29] or by unsupervised clustering analysis of multidimensional behavioral variance [30, 31]. The latter approach may allow to capture the complex genetic and behavioral heterogeneity of substance use disorders in a translationally relevant way. Extended access self-administration models, thus, serve as a key platform for capturing multiple addiction-relevant domains, while generating large datasets, particularly in large-scale GWAS studies, which require efficient data collection, processing, storage, and sharing. An automated pipeline can streamline data management to improve capabilities and keep up with high-throughput demands.

### Preprocessing of operant data with timestamps into spreadsheets

**Fig. 2:**
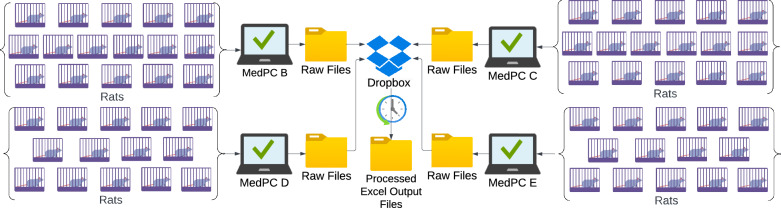
Setup of the operant chamber testing system and processing of the data. Max 16 operant chambers are controlled by a computer with MedPC software through an interface module. After every session, each computer generates 1 TXT file with the combined session data for all the boxes it controls, which is saved into a designated Raw Files folder on Dropbox. From there each TXT file is converted automatically into a more convenient Excel output file that is saved into a designated folder on Dropbox using Microsoft Task Scheduler running the GetOperant tool.

All operant self-administration data (drug infusions, active and inactive lever presses, under FR1, PR, and shock experiments) are automatically recorded with precise timestamps by proprietary software that comes with the operant chambers. The PARC system employs the Med Associated equipment and software suite, a widely used standard in behavioral neuroscience. Up to 16 operant chambers can be controlled by one interface module and computer. For testing 60 rats simultaneously, 60 operant chambers are managed by 4 dedicated computers (Fig. 2). At the end of each operant session, every computer generates a raw data file containing session data for its respective chambers (Suppl. File 1), saved in a dedicated folder on Dropbox. The raw files follow a standard but non-intuitive format with letters associated to event counts (e.g., B for lever presses), bin-count arrays (e.g., W for drug infusions per 5 min bins), and time arrays (e.g., Y for timestamps of active lever presses) in a TXT format, which is not directly interpretable or suitable for analysis. Historically, experimenters manually transferred total infusion and lever data to notebooks and spreadsheets. Alternatively, a Med-PC to Excel Data Transfer utility software “MPC2XL” is available to simplify the export of TXT files to Excel directly. Nevertheless, both approaches require experimenter effort, are error-prone, and are typically heterogeneous between experimenters. To eliminate this bottleneck, we implemented the open-source extraction tool for Med-PC data, GEToperant [11], to automatically process info from raw session files (including animal ID, session start and end date and time, number of drug infusions, active, and inactive lever presses) into a custom Excel output file (Suppl. File 2) saved in a dedicated Dropbox folder. Importantly, without any additional effort, timestamps are also extracted for each behavioral event to enable time-resolved analysis.

### Integration with other experimental information through homogenized spreadsheets

**Fig. 3:**
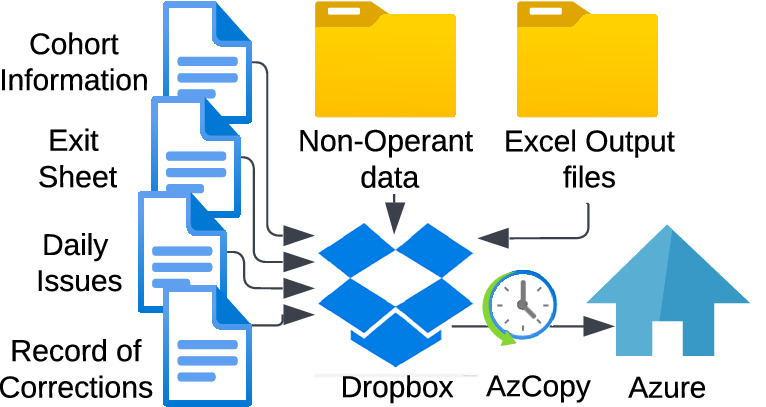
Summary of all the input data collected on Dropbox for upload to Azure. Data include the processed Excel output files from the operant sessions (see Fig. 3), Cohort Information file with details about the subjects and the performed experiments, Exit file with excluded animals, Daily Issues file with experimenter notes on the animals and operant sessions, and other non-operant experimental data like Tail Immersion (TI), Von Frey (VF), and Bottle Brush tests. All files are uploaded daily to Azure through AzCopy with Microsoft Task Scheduler, except for the Excel output files that are uploaded only once, but updated when listed in the Corrections Record file.

In addition to operant data, all other experimental data are uploaded using standardized Excel templates to dedicated Dropbox folders (Fig. 3). The entry point of animals, each with their unique RFID, in the pipeline is through a row in the Cohort Information file with additional metadata (animal ID, RFID, sex, treatment group; Suppl. File 3). Experimenter notes are essential to complement the automated upload of raw operant session data and identify or exclude faulty sessions. This operation is facilitated by the Daily Issues file in which a coded issue is listed per row for a specified animal and session (Suppl. File 4). Similarly, it is essential to track animals that get excluded from the study through an Exit file (Suppl. File 5). Besides identifying till which point data can be retained for this animal (last good session), it can also track completion of the behavioral testing for AI calculation and status of tissue collection for genotyping and biobanking. Non-operant behavioral tests are logged in dedicated template files for Von Frey (Suppl. File 6), tail immersion (Suppl. File 7), and bottle brush (Suppl. File 8) assays. Finally, to allow for efficient data transfer, a Corrections Record file is maintained to track modified files requiring re-upload from Dropbox to Azure (see next section). Harmonized formats ensure consistency, traceability, and downstream integration through linkage by RFID across all data, including session-level decisions, and animal-level metadata.

### Combining all data in a relational database

**Fig. 4:**
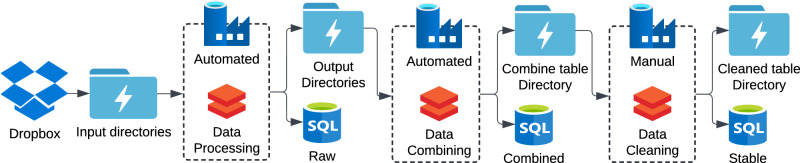
Data flow through 3 databases (raw, combined, and stable) in the Azure environment. The pipeline leverages Data Lake storage (thunderbolt folder icons), Data Factory automated processing (blue factory icons), Databricks with Python script for data manipulation (orange bricks icons), and SQL databases (SQL icons).

All behavioral and metadata files are ultimately integrated into a secure Azure-based relational database (Fig. 4). Azure, Microsoft’s cloud computing platform, provides optimized services for this task: Data Lake for data storage, Data Factory for orchestrating automated workflow, Databricks for scalable data manipulation using Python, and SQL databases for structured data queries. Dropbox is connected to Azure for seamless data transfer. All uploaded data files are parsed into separate data tables by type (Raw database; Suppl. List 1). These data tables are then joined using RFID into a single, unified table that excludes data from sessions discarded in the daily issues file or after the last good session in the exit file (Raw Combined database; Suppl. List 2). At intervals, typically in between cohorts, the raw table is transformed into a curated database through outlier removal, missing data imputation, and the calculation of dependent variables (Stable database; Suppl. List 3). The curated combined table is static to ensure integrity in case of pipeline disruptions.

### Automated and on-request data output and visualization

**Fig. 5:**
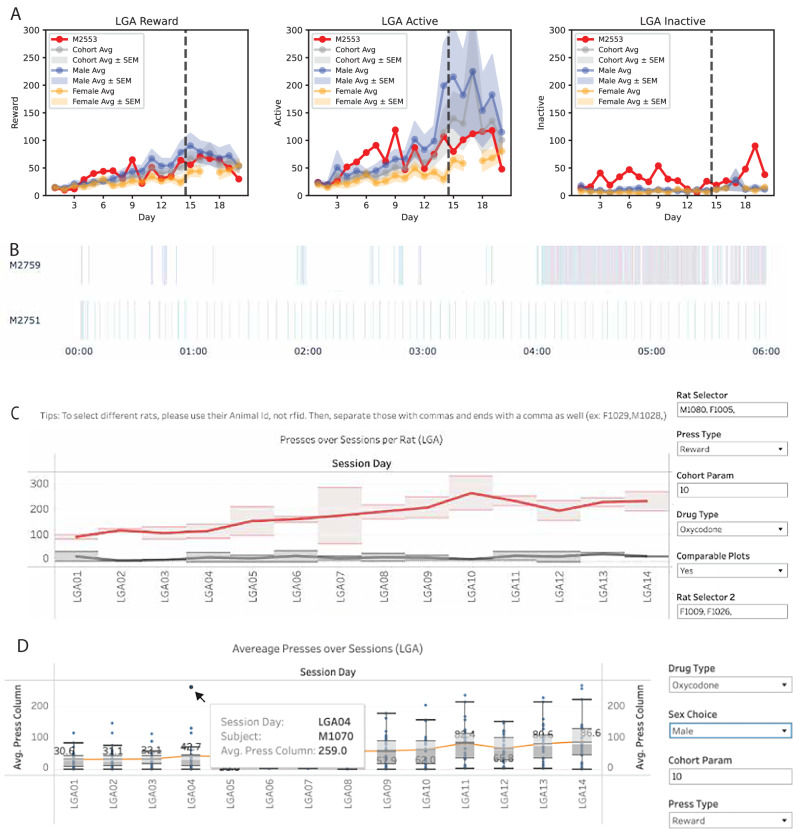
Example screenshots of data visualization applications. **A** Behavioral traces: subsection of an individual report showing daily evolution of the total drug infusions (left), active (middle) and inactive (right) lever presses during LgA for an animal (red) compared to cohort (gray), male (blue) and female (orange) averages and SEM. Any data issues that prevent proper processing of the data will be visibly missing, like the female data of LgA17. **B** Online tool for Excel output files: Esterline of time stamps for lever presses: active (blue), inactive (red), timeout (purple) presses and drug infusions (green) of 2 animals self-administering cocaine. M2751 shows high and regular intake. M2759 shows low irregular intake initially that transitions into very high pressing of both the active and inactive levers around ~4 h. **C,** **D** Tableau visualization: plotting the evolution of self-administration during LgA of 2 separate groups of animals (**C**) or of an entire cohort with easy identification of outliers (**D**).

Well-organized data, accessible on the cloud, allow for streamlined automated summary reports and data visualization, which can be provided in real-time as feedback to the experimenters. Detection of anomalies in an animal’s behavioral trend can signal hardware or health issues to check on and timely address. To aid in this assessment, all notes and collected data, both operant and non-operant, for each animal are graphically represented relative to its own history and the cohort average in a cumulative individual report (Fig. 5A, S3). This PDF is updated daily, so the graphs build over the experiment into a neatly organized animal behavioral identification page. The cohort operant total values themselves (total session infusions, active and inactive lever presses) are also automatically numerically summarized for all animals and sessions in an Excel behavior file (Suppl. File 9) with daily notes and animal exclusions, reminiscent of previous (semi-)manually maintained files. An additional advantage of these automated summaries is that all issues with data processing, like naming mistakes or missing files, will be signaled through data gaps in the report. When an experimenter might be aware of a session issue (like finding a disconnected animal at the end of a session), quick visualization of time-resolved behavior can help to determine when the issue occurred and, thus, support a decision on whether to discard or keep the data. To aid in this assessment, an online tool was developed that ingests Excel output files to generate interactive, timestamp-derived plots of selected animals and variables. One plot displays the timestamps of the various operant session events (drug infusions, active, inactive, and time-out lever presses) per animal across the session duration (Fig. 5B). Another plot represents the distribution of the inter-infusion intervals, calculated as the time between successive drug infusion timestamps in a histogram. Of course, all data are also organized in the database, of which a CSV backup is saved daily to Dropbox. For easy data investigation, the database is linked with Tableau [13] through a graphic user interface (GUI) with custom templates for data classification for insight in the dataset, variable distributions for insight in data variability, correlation of variables for insight in the relation between behaviors, other custom subgroup comparisons (Fig. 5C), and behavior evolution for insights in trends, like escalation across LgA (Fig. 5D).

## Discussion

We developed and deployed an automated data pipeline to manage, integrate, and visualize high-throughput behavioral data generated from large-scale phenotyping of addiction-like behaviors in rats. This system enabled the structured processing of raw operant data, other non-operant data, and associated metadata into a unified relational database, while also producing user-friendly real-time visual outputs for quality control and experiment oversight.

Traditional approaches to preclinical behavioral data handling rely on labor-intensive, error-prone (semi-)manual transcription and spreadsheet assembly, often differing between experimenters, cohorts, or timepoints, which complicates data integration, sharing, and analysis. In contrast, our pipeline incorporates automated extraction, standardized file formatting, cloud-based processing, and structured outputs. Automation avoids backlogged data, reduces experimenter workload and error, and provides opportunities for streamlining instant data processing with online access, quality checks, and data visualization, enabling real-time adjustments during ongoing experiments. Standardized tracking of daily issues and experimenter decisions further supports transparency. Moreover, the automated pipeline allows for retention of more information, such as event timestamps, for detailed analysis of time-resolved behavior within operant sessions and the characterization of patterns. As an added benefit, the approach thus enhances reproducibility, facilitates data exchange between researchers, and ensures existing valuable data are fully leveraged for scientific discovery and translatable progress toward clinical trials.

Dropbox linked to Microsoft Azure was chosen as the basis for the pipeline for accessibility, integration, and flexibility. Cloud-based solutions make the data accessible from anywhere at any time. Dropbox is easily accessible and well-integrated with access from every computer in the lab. Azure components AzCopy, Data Lake, Data Factory, Databricks, and SQL database are convenient for building secure, automated workflows. The initial set-up requires coding and data engineering expertise, but routine use of Azure pipeline to process standard Excel and CSV files from Dropbox is intuitive and generally user-friendly. Moreover, the infrastructure scales flexibly to meet changing demands, offering reliable performance without the need for local servers. Despite the fee for use, the overall cost is low considering the benefits.

This automated system was developed to support and has drastically improved high-throughput phenotyping capabilities for oxycodone and cocaine addiction-like behaviors in HS rats, by efficiently and reproducibly processing thousands of data files from over 2000 HS rats. While this implementation was optimized for this purpose, the general architecture can be adapted to other behavioral tasks, particularly when using operant chambers. The implementation of a more generalized and flexible design through minor modifications to data extraction rules and templates, enabling easier deployment across diverse behavioral paradigms and labs, is an opportunity for improvement in the future. Other future extensions may include integration with video tracking systems [32], biobanking inventories [1], as well as results from studies with Biobank samples [33–39].

In conclusion, here, we present our approach to automate data management, storage, processing, visualization, and sharing. Use of an automated pipeline simplifies processing, storing, analyzing, and sharing large amounts of data with minimal experimenter effort to obtain maximal data quality. This approach has enhanced PARC capabilities for robust and replicable characterization of addiction-like behaviors and accelerated the pace of discovery. Given the benefits of using an automated data pipeline, which also apply to smaller projects with operant behavior phenotyping, we share our established and optimized working approach to simplify the implementation process for others.

## Supplementary information

Supplemental Material

Supplemental File 2

Supplemental File 3

Supplemental File 4

Supplemental File 5

Supplemental File 6

Supplemental File 7

Supplemental File 8

Supplemental File 9

Supplemental File 1

## Data Availability

Pipeline Sample Code can be found on GitHub: https://github.com/3c-lab/operant-data-pipeline and has been published on Zenodo: https://zenodo.org/records/17058157 [40], where future release updates will be tracked. [Where relevant in the manuscript, files and subfolders from the repository were referenced between square brackets].
