# Supplementary material for: Automated pipeline for operant behavior phenotyping for high-throughput data management, processing, and visualization
Source: NPP Digit Psychiatry Neurosci. 2025 Oct 24;3:25. doi: 10.1038/s44277-025-00046-9 (PMC12624926; doi:10.1038/s44277-025-00046-9)
Supplement: Supplementary file 1 — Supplemental Material [file 44277_2025_46_MOESM1_ESM.pdf]

Supplemental Material for "Automated pipeline for operant behavior phenotyping for high-throughput data management, processing, and visualization."

Supplementary Materials and Methods

Animals, Drugs, and Behavioral Characterization: Animals undergo jugular vein catheterization for intravenous (*i.v.*) drug self-administration. Lever pressing on the active lever presses triggers a drug infusion paired with a cue light, followed by a 20-second timeout period during which additional presses have no consequence. The inactive lever has no consequence. Sessions are conducted on a fixed ratio 1 (FR1) schedule of reinforcement.

Cocaine Protocol:

- **Short Access (ShA):** 10 sessions, 2 hours/day, 500 µg/kg/infusion
- **Long Access (LgA):** 14 sessions, 6 hours/day, same dose
- **Progressive Ratio (PR):** Conducted after ShA, LgA, and shock sessions
- **Shock Test:** Session pairing drug infusions with mild footshock to assess compulsivity- like behavior
- **Irritability Test:** Bottlebrush test at baseline and after LgA
- The Addiction index (AI) is calculated as average of the Z-scores of escalation (intake during last 3 days of LgA), motivation (intake during PR) and compulsivity (intake despite footshock)

Oxycodone Protocol:

- **Short Access (ShA):** 4 sessions, 2 hours/day, 150 µg/kg/infusion
- **Long Access (LgA):** 14 sessions, 12 hours/day, same dose
- **Progressive Ratio (PR):** Conducted after ShA and LgA, and to test effects of FDA approved treatments: buprenorphine, methadone, and naltrexone, vs vehicle
- **Hyperalgesia:** Von Frey test at baseline and after LgA
- **Analgesia/Tolerance:** Tail immersion test before ShA (with and without oxycodone) and after LgA (with oxycodone)
- The Addiction index (AI) is calculated as average of the Z-scores of of escalation, motivation, hyperalgesia (reduced pain threshold during withdrawal), and tolerance (reduced analgesia)

Exclusion Criteria and Health Checks: Catheter patency is confirmed at the end of LgA via a short-acting anesthetic (Brevital). Animals failing to respond are excluded.

RFID and Tracking Each rat receives a unique subcutaneous radio frequency identification (RFID) tag at study onset, which is scanned before behavior tests and saved with the spleen for genomic processing. Subject ID and RFID are used across all data files for consistent identification and integration.

Data: Each animal has basic info (contained in a Cohort Information file), performs >30 operant sessions (4-10 ShA sessions, 14-20 LgA sessions, 3-6 PR sessions, and 0-2 shock sessions; contained in daily Excel output files derived from MedPC TXT files), in addition to other behavioral tests (with results listed in standardized Excel files) and has experimenter notes on daily issues, exclusions or replacements (contained in Daily Issues and Exit files). Together, this amounts to >100,000 files to keep track of and link.

Additional details can be found in previous publications and the public George lab protocol repository on protocols.io (https://www.protocols.io/workspaces/george-lab)

Supplemental Figures

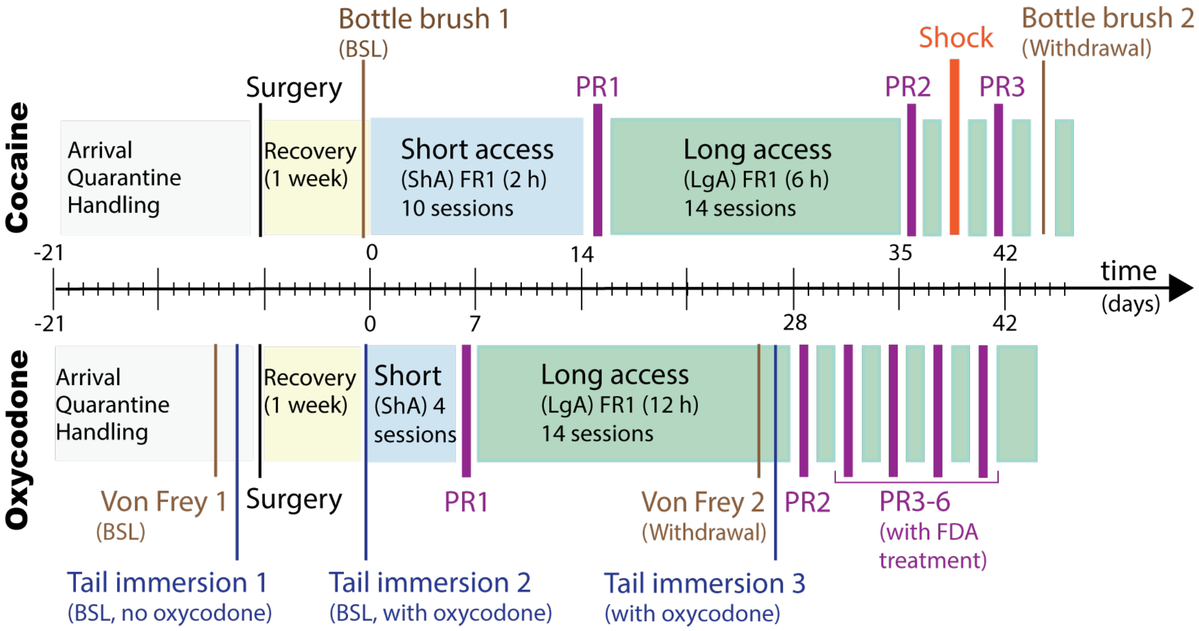

***Figure S1. Experimental timeline of standard operating procedures for addiction-like behavior phenotyping** of cocaine (top) and oxycodone (bottom)**.** The timeline starts 1 week following recovery from jugular vein surgery with eight-to ten-week-old rats. The behavioral measures include self-administration under fixed ratio 1 (FR1) with short (ShA) and long (LgA) access, hyperalgesia using the von Frey test (for oxycodone) at LgA compared with baseline (BSL), irritability-like behavior using the bottlebrush test (for cocaine) at LgA compared with BSL, opioid-induced analgesia, and tolerance using the tail immersion test (for oxycodone) before ShA and after LgA with oxycodone on board relatively compared with BSL and BSL with oxycodone, motivation with progressive ratio (PR) test (for both oxycodone and cocaine) after ShA and LgA (and after shock for cocaine only), compulsivity with FR1 using contingent footshocks (for cocaine) and the effect of FDA-approved treatments after LgA on PR tests (for oxycodone). Adjusted from Carrette et. al. 2021 eNeuro.*

rfid	subject	room	cohort	trial_id	drug	box	start_time	end_time	start_date	end_date	active_lever_presses	inactive_lever_presses	reward_presses	timeout_presses
**933000320499823**	F1817	MTF134C	18	LGA01	cocaine	1	08:59:52	15:32:58	2021-09-15	2021-09-15	19	3	16	3
**933000320499680**	F1818	MTF134C	18	LGA01	cocaine	2	08:59:54	15:32:58	2021-09-15	2021-09-15	175	0	113	62
**933000320499811**	F1819	MTF134C	18	LGA01	cocaine	3	08:59:56	15:32:58	2021-09-15	2021-09-15	104	9	62	42
**933000320499684**	F1820	MTF134C	18	LGA01	cocaine	4	09:00:00	15:32:58	2021-09-15	2021-09-15	18	4	7	11
**933000320499809**	F1821	MTF134C	18	LGA01	cocaine	5	09:00:03	15:32:58	2021-09-15	2021-09-15	86	9	62	24
**933000320499807**	F1822	MTF134C	18	LGA01	cocaine	6	09:00:07	15:32:58	2021-09-15	2021-09-15	85	0	49	36
**933000320499683**	F1823	MTF134C	18	LGA01	cocaine	7	09:00:10	15:32:58	2021-09-15	2021-09-15	184	0	146	38
**933000320499820**	F1824	MTF134C	18	LGA01	cocaine	8	09:00:12	15:32:58	2021-09-15	2021-09-15	119	6	82	37
**933000320500013**	F1825	MTF134C	18	LGA01	cocaine	9	09:00:14	15:32:58	2021-09-15	2021-09-15	18	16	18	0
**933000320500025**	F1826	MTF134C	18	LGA01	cocaine	10	09:00:16	15:32:58	2021-09-15	2021-09-15	68	5	56	12
**933000320500017**	F1827	MTF134C	18	LGA01	cocaine	11	09:00:18	15:32:58	2021-09-15	2021-09-15	81	8	42	39
**933000320499961**	F1828	MTF134C	18	LGA01	cocaine	12	09:00:20	15:32:58	2021-09-15	2021-09-15	169	0	101	68
**933000320500007**	F1829	MTF134C	18	LGA01	cocaine	13	09:00:23	15:32:58	2021-09-15	2021-09-15	123	9	83	40
**933000320499970**	F1830	MTF134C	18	LGA01	cocaine	14	09:00:26	15:32:58	2021-09-15	2021-09-15	60	1	40	20
**933000320499930**	M1851	MTF134C	18	LGA01	cocaine	15	09:00:32	15:32:58	2021-09-15	2021-09-15	79	0	73	6
**933000320500442**	M1852	MTF134C	18	LGA01	cocaine	16	09:00:35	15:32:58	2021-09-15	2021-09-15	77	8	73	4

rfid	rat	cohort	experiment_group	drug_group	sex	arrival_date	age_at_arrival	uv	brevital	brevital_date	brevital_technicians	lga_15_date	lga_16_date
**933000120117306**	F19	1	Drug	Cocaine	Female	2017-06-29 00:00:00.0000000						2017-09-07 00:00:00.0000000	2017-09-12 00:00:00.0000000
**933000120117307**	M61	1	Drug	Cocaine	Male	2017-06-29 00:00:00.0000000						2017-09-07 00:00:00.0000000	2017-09-12 00:00:00.0000000
**933000120117308**	M66	1	Drug	Cocaine	Male	2017-06-29 00:00:00.0000000						2017-09-07 00:00:00.0000000	2017-09-12 00:00:00.0000000
**933000120117309**	F25	1	Naive	Cocaine	Female	2017-06-29 00:00:00.0000000							
**933000120117310**	F24	1	Naive	Cocaine	Female	2017-06-29 00:00:00.0000000							
**933000120117311**	F21	1	Drug	Cocaine	Female	2017-06-29 00:00:00.0000000						2017-09-07 00:00:00.0000000	2017-09-12 00:00:00.0000000
**933000120117312**	F20	1	Drug	Cocaine	Female	2017-06-29 00:00:00.0000000						2017-09-07 00:00:00.0000000	2017-09-12 00:00:00.0000000
**933000120117313**	F26	1	Drug	Cocaine	Female	2017-06-29 00:00:00.0000000						2017-09-07 00:00:00.0000000	2017-09-12 00:00:00.0000000
**933000120117315**	M96	1	Naive	Cocaine	Male	2017-06-29 00:00:00.0000000							
**933000120117318**	M54	1	Drug	Cocaine	Male	2017-06-29 00:00:00.0000000						2017-09-07 00:00:00.0000000	2017-09-12 00:00:00.0000000
**933000120117320**	M58	1	Drug	Cocaine	Male	2017-06-29 00:00:00.0000000						2017-09-07 00:00:00.0000000	2017-09-12 00:00:00.0000000
**933000120117321**	M55	1	Drug	Cocaine	Male	2017-06-29 00:00:00.0000000						2017-09-07 00:00:00.0000000	2017-09-12 00:00:00.0000000
**933000120117322**	M62	1	Drug	Cocaine	Male	2017-06-29 00:00:00.0000000							
**933000120117323**	F23	1	Drug	Cocaine	Female	2017-06-29 00:00:00.0000000						2017-09-07 00:00:00.0000000	2017-09-12 00:00:00.0000000
**933000120117324**	M65	1	Drug	Cocaine	Male	2017-06-29 00:00:00.0000000						2017-09-07 00:00:00.0000000	2017-09-12 00:00:00.0000000
**933000120117325**	M59	1	Drug	Cocaine	Male	2017-06-29 00:00:00.0000000						2017-09-07 00:00:00.0000000	2017-09-12 00:00:00.0000000
**933000120117326**	M69	1	Drug	Cocaine	Male	2017-06-29 00:00:00.0000000						2017-09-07 00:00:00.0000000	2017-09-12 00:00:00.0000000
**933000120117327**	M70	1	Naive	Cocaine	Male	2017-06-29 00:00:00.0000000							
**933000120117329**	F22	1	Drug	Cocaine	Female	2017-06-29 00:00:00.0000000						2017-09-07 00:00:00.0000000	2017-09-12 00:00:00.0000000
**933000120117330**	M95	1	Naive	Cocaine	Male	2017-06-29 00:00:00.0000000							

rfid	measurement_name	measurement_value	drug_group	cohort	measure_number	date_measured	technician
**933000120117306**	feces		cocaine	1	1	2017-08-01 00:00:00.0000000	
**933000120117306**	feces		cocaine	1	2	2017-09-01 00:00:00.0000000	
**933000120117306**	feces		cocaine	1	3		
**933000120117306**	feces		cocaine	1	4		
**933000120117306**	urine		cocaine	1	1		
**933000120117306**	urine		cocaine	1	2		
**933000120117306**	weight	167	cocaine	1	1	2017-07-25 00:00:00.0000000	
**933000120117306**	weight	177	cocaine	1	2	2017-08-04 00:00:00.0000000	
**933000120117306**	weight	187	cocaine	1	3	2017-08-11 00:00:00.0000000	
**933000120117306**	weight	197	cocaine	1	4	2017-08-18 00:00:00.0000000	
**933000120117306**	weight	211	cocaine	1	5	2017-08-30 00:00:00.0000000	
**933000120117306**	weight	215	cocaine	1	6	2017-09-08 00:00:00.0000000	
**933000120117306**	weight		cocaine	1	7		
**933000120117306**	weight		cocaine	1	8		
**933000120117306**	weight		cocaine	1	9		
**933000120117306**	weight		cocaine	1	10		
**933000120117307**	feces		cocaine	1	1	2017-08-01 00:00:00.0000000	
**933000120117307**	feces		cocaine	1	2	2017-09-01 00:00:00.0000000	
**933000120117307**	feces		cocaine	1	3		
**933000120117307**	feces		cocaine	1	4		
**933000120117307**	urine		cocaine	1	1		
**933000120117307**	urine		cocaine	1	2		
**933000120117307**	weight	259	cocaine	1	1	2017-07-24 00:00:00.0000000	
**933000120117307**	weight	244	cocaine	1	2	2017-08-04 00:00:00.0000000	
**933000120117307**	weight	264	cocaine	1	3	2017-08-11 00:00:00.0000000	
**933000120117307**	weight	287	cocaine	1	4	2017-08-18 00:00:00.0000000	
**933000120117307**	weight	301	cocaine	1	5	2017-08-30 00:00:00.0000000	
**933000120117307**	weight	316	cocaine	1	6	2017-09-08 00:00:00.0000000	

Figure S2. Example screenshots of the processed files in the Azure pipeline. *CSV file following processing of the Excel output files with the Azure pipeline. Each row contains the information of a specific session of a specific rat, the session summary data, and associated timestamps for lever presses and drug infusions (Top). CSV files following the processing of the Cohort Information files with the Azure pipeline. Each row contains the information of a specific rat, with subject information (Middle) and measurements (Bottom).*

Rat: M2553 RFID: 933000321073389

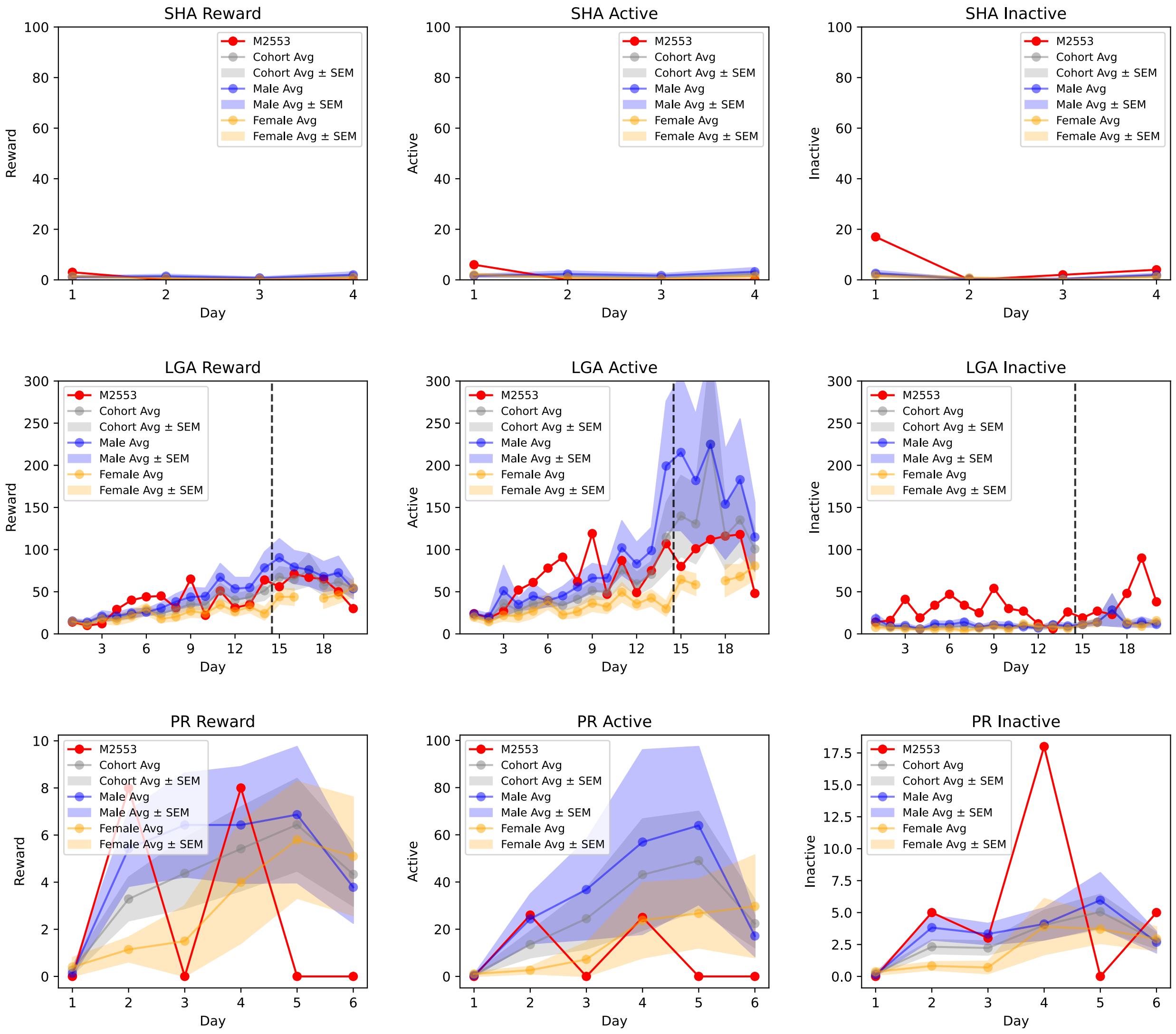

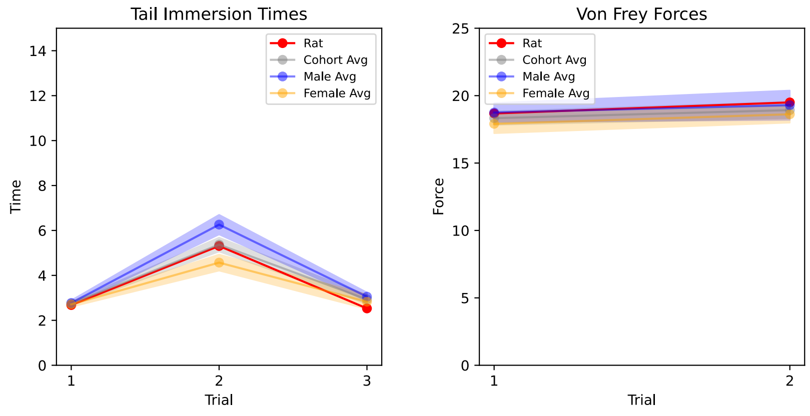

RatID : M2553
RFID : 933000321073389

Issues :
None

Notes :
None

ExitCode : None
ExitNote : None

Figure S3. Example page from the behavioral trace PDF file. *Behavioral performance across all operant phases and additional tests is shown for a representative rat**,** M2553 tested for oxycodone addiction-like behaviors, as identified on the top of the file. Line graphs depict the individual rat's behavior compared to cohort average, male average, and female average, with shaded areas representing standard error of the mean (SEM) for ShA (1st row), LgA (2nd row), and PR (3rd row) self-administration, tail immersion (4th row, left) and Von Frey test (4th row, middle). Other relevant data to help with the interpretation of the behavioral data on animal char*

Supplemental Related Manuscript File - Legends

1. Example of a raw MedPC output TXT file, which is saved directly in a designated directory on Dropbox following the operant session.
2. Example of a customized Excel output file, which is automatically generated for each MedPC TXT file using the GetOperant tool and saved in a designated Dropbox directory. Within each file, the first ‘index column’ defines the measurements (session summary data for lever presses and drug infusions, and the associated timestamps), the other columns contain the specified information for a specific rat, each.
3. Example of a Cohort Information file, which is saved directly in a designated directory on Dropbox
4. Example of a Daily Issues file, which is updated daily with experimenter notes in a designated directory on Dropbox
5. Example of an Exit file, which is updated daily with experimenter notes in a designated directory on Dropbox
6. Example of a Standardized Excel file for hyperalgesia measurements using the Von Frey test saved in its designated directory on Dropbox
7. Example of a Standardized Excel file for analgesia and tolerance measurements using tail immersion tests saved in its designated directory on Dropbox
8. Example of a Standardized Excel file for irritability-like behavior using bottle brush tests saved in its designated directory on Dropbox
9. Example of a Behavior file, a summary of the operant data per cohort, which is automatically updated daily with the new processed data and comments from the Cohort Information, Daily Issues, and Exit files.

Supplemental Variable lists

Supplemental List 1. Variables extracted in the separate tables of the SQL database

- **From the Excel output files**
  - **Trial_sha**
    - rfid: bigint
    - subject: varchar
    - room: varchar
    - cohort: int
    - trial_id: varchar
    - drug: varchar
    - box: int
    - start_time: time
    - end_time: time
    - start_date: date
    - end_date: date
    - active_lever_presses: int
    - inactive_lever_presses: int
    - reward_presses: int
    - timeout_presses: int
    - inactive_timestamps: text
    - reward_timestamps: text
    - timeout_timestamps: text
  - **Trial_lga**
    - rfid: bigint
    - subject: varchar
    - room: varchar
    - cohort: int
    - trial_id: varchar
    - drug: varchar
    - box: int
    - start_time: time
    - end_time: time
    - start_date: date
    - end_date: date
    - active_lever_presses: int
    - inactive_lever_presses: int
    - reward_presses: int
    - timeout_presses: int
    - inactive_timestamps: text
    - reward_timestamps: text
    - timeout_timestamps: text
  - **Trial_pr**
    - rfid: bigint
    - subject: varchar
    - room: varchar
    - cohort: int
    - trial_id: varchar

- drug: varchar
- box: int
- start_time: time
- end_time: time
- start_date: date
- end_date: date
- breakpoint: int
- last_ratio: int
- ratios: text
- active_lever_presses: int
- inactive_lever_presses: int
- reward_presses: int
- timeout_presses: int
- **Trial_shock**
  - rfid: bigint
  - subject: varchar
  - room: varchar
  - cohort: int
  - trial_id: varchar
  - drug: varchar
  - box: int
  - start_time: time
  - end_time: time
  - start_date: date
  - end_date: date
  - total_active_lever_presses: int
  - total_inactive_lever_presses: int
  - total_shocks: int
  - total_reward: int
  - rewards_after_first_shock: int
  - rewards_got_shock: text
  - Reward_timestamps:

- **From the Daily Issues**
  - **Trial_note**
    - rfid: bigint
    - subject: varchar
    - cohort: int
    - sex: varchar
    - drug: varchar
    - experiment_group: varchar
    - trial_id: varchar
    - start_date: date
    - code: varchar
    - to_do: varchar
    - Note: text

- **From the other experiment files**

- **Von_frey**
    - rfid: bigint
    - subject: varchar
    - cohort: int
    - sex: varchar
    - drug: varchar
    - experiment_group: varchar
    - vf1_right_force_1: String
    - vf1_right_force_2: String
    - vf1_right_force_3: String
    - vf1_right_force_avg: String
    - vf1_right_time_1: String
    - vf1_right_time_2: String
    - vf1_right_time_3: String
    - vf1_right_avg: String
    - vf1_left_force_1: String
    - vf1_left_force_2: String
    - vf1_left_force_3: String
    - vf1_left_force_avg: String
    - vf1_left_time_1: String
    - vf1_left_time_2: String
    - vf1_left_time_3: String
    - vf1_left_avg: String
    - von_frey_1_date: String
    - vf2_right_force_1: String
    - vf2_right_force_2: String
    - vf2_right_force_3: String
    - vf2_right_force_avg: String
    - vf2_right_time_1: String
    - vf2_right_time_2: String
    - vf2_right_time_3: String
    - vf2_right_avg: String
    - vf2_left_force_1: String
    - vf2_left_force_2: String
    - vf2_left_force_3: String
    - vf2_left_force_avg: String
    - vf2_left_time_1: String
    - vf2_left_time_2: String
    - vf2_left_time_3: String
    - vf2_left_avg: String
    - von_frey_2_date: String
    - von_frey_1_force: String
    - von_frey_1_time: String
    - von_frey_2_force: String
    - von_frey_2_time: String
    - von_frey_difference_force: String

- **Tail_immersion**

- rfid: bigint
- subject: varchar
- cohort: int
- sex: varchar
- experiment_group: varchar
- drug: varchar
- tail_immersion_1_time: float
- tail_immersion_2_time: float
- tail_immersion_3_time: float
- tail_immersion_difference_tolerance: float
- tail_immersion_1_date: date
- tail_immersion_2_date: date
- tail_immersion_3_date: date

- **Irritability**
  - rfid: bigint
  - subject: varchar
  - cohort: int
  - sex: varchar
  - group: varchar
  - def_bsl_irr_1: String
  - agg_bsl_irr_1: String
  - irr_bsl_tot_1: String
  - irr_bsl_scorer_1: String
  - def_bsl_irr_2: String
  - agg_bsl_irr_2: String
  - irr_bsl_total_2: String
  - irr_bsl_scorer_2: String
  - def_bsl_irr_3: String
  - agg_bsl_irr_3: String
  - irr_bsl_total_3: String
  - irr_bsl_scorer_3: String
  - def_bsl_ave: String
  - agg_bsl_ave: String
  - total_bsl_ave: String
  - def_drug_irr_1: String
  - agg_drug_irr_1: String
  - irr_drug_tot_1: String
  - irr_drug_scorer_1: String
  - def_drug_irr_2: String
  - agg_drug_irr_2: String
  - irr_drug_total_2: String
  - irr_drug_scorer_2: String
  - def_drug_irr_3: String
  - agg_drug_irr_3: String
  - irr_drug_total_3: String
  - irr_drug_scorer_3: String
  - def_drug_ave: String

        - agg_drug_ave: String
        - total_drug_ave: String
        - diff_ave_def: String
        - diff_ave_agg: String
        - diff_ave_total: String

- **From the Cohort Information file**
    - **Subject**
        - rfid: String
        - rat: String
        - cohort: String
        - experiment_group: String
        - drug_group: String
        - sex: String
        - arrival_date: String
        - age_at_arrival: String
        - uv: String
        - brevital: String
        - brevital_date: String
        - brevital_technicians: String
        - lga_15_date: String
        - lga_16_date: String
        - lga_17_date: String
        - lga_18_date: String
        - lga_19_date: String
        - lga_20_date: String
        - age_at_lga: String
        - long_access_start_date: String
        - long_access_end_date: String
        - age_at_sha: String
        - short_access_start_date: String
        - short_access_end_date: String
        - pre_shock_date: String
        - shock_1_date: String
        - shock_2_date: String
        - shock_3_date: String
        - female_swab_1_technicians: String
        - female_swab_1_date: String
        - female_swab_1_analysis: String
        - female_swab_2_technicians: String
        - female_swab_2_date: String
        - female_swab_2_analysis: String
        - female_swab_3_technicians: String
        - female_swab_3_date: String
        - female_swab_3_analysis: String
        - irritability_1_technicians: String
        - irritability_1_date: String
        - irritability_2_technicians: String

- irritability_2_date: String
- von_frey_1_technicians: String
- von_frey_1_date: String
- von_frey_2_technicians: String
- von_frey_2_date: String
- tail_immersion_1_technicians: String
- tail_immersion_1_date: String
- tail_immersion_2_technicians: String
- tail_immersion_2_date: String
- tail_immersion_3_technicians: String
- tail_immersion_3_date: String
- lga_pre_treatment_1_date: String
- lga_pre_treatment_2_date: String
- lga_pre_treatment_3_date: String
- lga_pre_treatment_4_date: String
- lga_post_treatment_1_date: String
- lga_post_treatment_2_date: String
- lga_post_treatment_3_date: String
- lga_post_treatment_4_date: String
- progressive_ratio_1_date: String
- progressive_ratio_2_date: String
- progressive_ratio_3_date: String
- treatment_1_date: String
- treatment_1_group: String
- treatment_1_start_time: String
- treatment_2_date: String
- treatment_2_group: String
- treatment_2_start_time: String
- treatment_3_date: String
- treatment_3_group: String
- treatment_3_start_time: String
- treatment_4_date: String
- treatment_4_group: String
- treatment_4_start_time: String
- coat_color: String
- date_of_birth: String
- date_of_eye_bleed: String
- date_of_ship: String
- date_of_wean: String
- age_at_dissection: String
- dissection_group: String
- dissection_date: String
- ear_punch: String
- group_pre_shock: String
- group_shock: String
- handled_by: String
- litter_number: String
- litter_size: String

- rack: String
- recatheter_surgeon: String
- recatheter_surgery_date: String
- shipping_box: String
- surgeon: String
- surgery_assist: String
- surgery_date: String
- age_at_surgery: String
- date_of_death: String
- days_of_experiment: String
- exit_day: String
- last_good_session: String
- exit_code: String
- complete: String
- tissue_collected: String
- exit_notes: String
- replaced_by: String
- decision_exit : text
- decision_issues: text

- **Measurement**
  - rfid: String
  - measurement_name: String
  - measurement_value: String
  - drug_group: String
  - cohort: String
  - measure_number: String
  - date_measured: String
  - technician: String

Supplemental List 2. Variables in the raw combined SQL database

- rfid: varchar(255),
- rat: VARCHAR(255),
- cohort: integer,
- experiment_group: VARCHAR(255),
- drug_group: VARCHAR(255),
- sex: VARCHAR(255),
- uv: varchar(255),
- exit_notes: text,
- shipping_box: VARCHAR(255),
- exit_code: varchar(255),
- tissue_collected: varchar(255),
- complete: varchar(255),
- dissection_group: VARCHAR(255),
- brevital: varchar(255),
- last_good_session: varchar(255),
- replaced_by: varchar(255),
- decision_exit : text
- decision_issues: text
- coat_color: VARCHAR(255),
- ear_punch: VARCHAR(255),
- litter_number: integer,
- litter_size: integer,
- rack: VARCHAR(255),
- female_swab_1_analysis: VARCHAR(255),
- female_swab_2_analysis: VARCHAR(255),
- female_swab_3_analysis: VARCHAR(255),
- reward_presses_SHA01: integer,
- reward_presses_SHA02: integer,
- reward_presses_SHA03: integer,
- reward_presses_SHA04: integer,
- reward_presses_SHA05: integer,
- reward_presses_SHA06: integer,
- reward_presses_SHA07: integer,
- reward_presses_SHA08: integer,
- reward_presses_SHA09: integer,
- reward_presses_SHA10: integer,
- active_lever_presses_SHA01: integer,
- active_lever_presses_SHA02: integer,
- active_lever_presses_SHA03: integer,
- active_lever_presses_SHA04: integer,
- active_lever_presses_SHA05: integer,
- active_lever_presses_SHA06: integer,
- active_lever_presses_SHA07: integer,
- active_lever_presses_SHA08: integer,
- active_lever_presses_SHA09: integer,
- active_lever_presses_SHA10: integer,

- timeout_presses_SHA01: integer,
- timeout_presses_SHA02: integer,
- timeout_presses_SHA03: integer,
- timeout_presses_SHA04: integer,
- timeout_presses_SHA05: integer,
- timeout_presses_SHA06: integer,
- timeout_presses_SHA07: integer,
- timeout_presses_SHA08: integer,
- timeout_presses_SHA09: integer,
- timeout_presses_SHA10: integer,
- inactive_lever_presses_SHA01: integer,
- inactive_lever_presses_SHA02: integer,
- inactive_lever_presses_SHA03: integer,
- inactive_lever_presses_SHA04: integer,
- inactive_lever_presses_SHA05: integer,
- inactive_lever_presses_SHA06: integer,
- inactive_lever_presses_SHA07: integer,
- inactive_lever_presses_SHA08: integer,
- inactive_lever_presses_SHA09: integer,
- inactive_lever_presses_SHA10: integer,
- reward_presses_LGA01: integer,
- reward_presses_LGA02: integer,
- reward_presses_LGA03: integer,
- reward_presses_LGA04: integer,
- reward_presses_LGA05: integer,
- reward_presses_LGA06: integer,
- reward_presses_LGA07: integer,
- reward_presses_LGA08: integer,
- reward_presses_LGA09: integer,
- reward_presses_LGA10: integer,
- reward_presses_LGA11: integer,
- reward_presses_LGA12: integer,
- reward_presses_LGA13: integer,
- reward_presses_LGA14: integer,
- active_lever_presses_LGA01: integer,
- active_lever_presses_LGA02: integer,
- active_lever_presses_LGA03: integer,
- active_lever_presses_LGA04: integer,
- active_lever_presses_LGA05: integer,
- active_lever_presses_LGA06: integer,
- active_lever_presses_LGA07: integer,
- active_lever_presses_LGA08: integer,
- active_lever_presses_LGA09: integer,
- active_lever_presses_LGA10: integer,
- active_lever_presses_LGA11: integer,
- active_lever_presses_LGA12: integer,
- active_lever_presses_LGA13: integer,
- active_lever_presses_LGA14: integer,

- timeout_presses_LGA01: integer,
- timeout_presses_LGA02: integer,
- timeout_presses_LGA03: integer,
- timeout_presses_LGA04: integer,
- timeout_presses_LGA05: integer,
- timeout_presses_LGA06: integer,
- timeout_presses_LGA07: integer,
- timeout_presses_LGA08: integer,
- timeout_presses_LGA09: integer,
- timeout_presses_LGA10: integer,
- timeout_presses_LGA11: integer,
- timeout_presses_LGA12: integer,
- timeout_presses_LGA13: integer,
- timeout_presses_LGA14: integer,
- active_lever_presses_lga15: integer,
- active_lever_presses_lga16: integer,
- active_lever_presses_lga17: integer,
- active_lever_presses_lga18: integer,
- active_lever_presses_lga19: integer,
- active_lever_presses_lga20: integer,
- active_lever_presses_lga21: integer,
- active_lever_presses_lga22: integer,
- active_lever_presses_lga23: integer,
- active_lever_presses_lga24: integer,
- active_lever_presses_lga25: integer,
- active_lever_presses_lga26: integer,
- active_lever_presses_lga27: integer,
- active_lever_presses_lga28: integer,
- timeout_presses_lga15: integer,
- timeout_presses_lga16: integer,
- timeout_presses_lga17: integer,
- timeout_presses_lga18: integer,
- timeout_presses_lga19: integer,
- timeout_presses_lga20: integer,
- timeout_presses_lga21: integer,
- timeout_presses_lga22: integer,
- timeout_presses_lga23: integer,
- timeout_presses_lga24: integer,
- timeout_presses_lga25: integer,
- timeout_presses_lga26: integer,
- timeout_presses_lga27: integer,
- timeout_presses_lga28: integer,
- inactive_lever_presses_lga15: integer,
- inactive_lever_presses_lga16: integer,
- inactive_lever_presses_lga17: integer,
- inactive_lever_presses_lga18: integer,
- inactive_lever_presses_lga19: integer,
- inactive_lever_presses_lga20: integer,

- inactive_lever_presses_lga21: integer,
- inactive_lever_presses_lga22: integer,
- inactive_lever_presses_lga23: integer,
- inactive_lever_presses_lga24: integer,
- inactive_lever_presses_lga25: integer,
- inactive_lever_presses_lga26: integer,
- inactive_lever_presses_lga27: integer,
- inactive_lever_presses_lga28: integer,
- von_frey_1_force: float,
- von_frey_1_time: float,
- von_frey_2_force: float,
- von_frey_2_time: float,
- von_frey_difference_force: float,
- tail_immersion_1_time: float,
- tail_immersion_2_time: float,
- tail_immersion_3_time: float,
- tail_immersion_difference_tolerance: float,
- def_bsl_ave: numeric,
- agg_bsl_ave: numeric,
- total_bsl_ave: numeric,
- def_drug_ave: numeric,
- agg_drug_ave: numeric,
- total_drug_ave: numeric,
- diff_ave_def: numeric,
- diff_ave_agg: numeric,
- diff_ave_total: numeric,
- reward_timestamps_sha01: text,
- reward_timestamps_sha02: text,
- reward_timestamps_sha03: text,
- reward_timestamps_sha04: text,
- reward_timestamps_sha05: text,
- reward_timestamps_sha06: text,
- reward_timestamps_sha07: text,
- reward_timestamps_sha08: text,
- reward_timestamps_sha09: text,
- reward_timestamps_sha10: text,
- active_timestamps_sha01: text,
- active_timestamps_sha02: text,
- active_timestamps_sha03: text,
- active_timestamps_sha04: text,
- active_timestamps_sha05: text,
- active_timestamps_sha06: text,
- active_timestamps_sha07: text,
- active_timestamps_sha08: text,
- active_timestamps_sha09: text,
- active_timestamps_sha10: text,
- timeout_timestamps_sha01: text,
- timeout_timestamps_sha02: text,

- timeout_timestamps_sha03: text,
- timeout_timestamps_sha04: text,
- timeout_timestamps_sha05: text,
- timeout_timestamps_sha06: text,
- timeout_timestamps_sha07: text,
- timeout_timestamps_sha08: text,
- timeout_timestamps_sha09: text,
- timeout_timestamps_sha10: text,
- inactive_timestamps_sha01: text,
- inactive_timestamps_sha02: text,
- inactive_timestamps_sha03: text,
- inactive_timestamps_sha04: text,
- inactive_timestamps_sha05: text,
- inactive_timestamps_sha06: text,
- inactive_timestamps_sha07: text,
- inactive_timestamps_sha08: text,
- inactive_timestamps_sha09: text,
- inactive_timestamps_sha10: text,
- reward_timestamps_lga01: text,
- reward_timestamps_lga02: text,
- reward_timestamps_lga03: text,
- reward_timestamps_lga04: text,
- reward_timestamps_lga05: text,
- reward_timestamps_lga06: text,
- reward_timestamps_lga07: text,
- reward_timestamps_lga08: text,
- reward_timestamps_lga09: text,
- reward_timestamps_lga10: text,
- reward_timestamps_lga11: text,
- reward_timestamps_lga12: text,
- reward_timestamps_lga13: text,
- reward_timestamps_lga14: text,
- active_timestamps_lga01: text,
- active_timestamps_lga02: text,
- active_timestamps_lga03: text,
- active_timestamps_lga04: text,
- active_timestamps_lga05: text,
- active_timestamps_lga06: text,
- active_timestamps_lga07: text,
- active_timestamps_lga08: text,
- active_timestamps_lga09: text,
- active_timestamps_lga10: text,
- active_timestamps_lga11: text,
- active_timestamps_lga12: text,
- active_timestamps_lga13: text,
- active_timestamps_lga14: text,
- timeout_timestamps_lga01: text,
- timeout_timestamps_lga02: text,

- timeout_timestamps_lga03: text,
- timeout_timestamps_lga04: text,
- timeout_timestamps_lga05: text,
- timeout_timestamps_lga06: text,
- timeout_timestamps_lga07: text,
- timeout_timestamps_lga08: text,
- timeout_timestamps_lga09: text,
- timeout_timestamps_lga10: text,
- timeout_timestamps_lga11: text,
- timeout_timestamps_lga12: text,
- timeout_timestamps_lga13: text,
- timeout_timestamps_lga14: text,
- inactive_timestamps_lga01: text,
- inactive_timestamps_lga02: text,
- inactive_timestamps_lga03: text,
- inactive_timestamps_lga04: text,
- inactive_timestamps_lga05: text,
- inactive_timestamps_lga06: text,
- inactive_timestamps_lga07: text,
- inactive_timestamps_lga08: text,
- inactive_timestamps_lga09: text,
- inactive_timestamps_lga10: text,
- inactive_timestamps_lga11: text,
- inactive_timestamps_lga12: text,
- inactive_timestamps_lga13: text,
- inactive_timestamps_lga14: text,
- ratios_pr01: text
- ratios_pr02: text
- ratios_pr03: text
- ratios_treatment01: text
- ratios_treatment02: text
- ratios_treatment03: text
- ratios_treatment04: text
- group_pre_shock: integer
- group_shock: integer
- rewards_got_shock_preshock: text
- rewards_got_shock_shock_v1: text
- rewards_got_shock_shock_v2: text
- rewards_got_shock_shock_v3: text
- reward_timestamps_preshock: text
- reward_timestamps_shock_v1: text
- reward_timestamps_shock_v2: text
- reward_timestamps_shock_v3: text
- reward_timestamps_sha11: text
- active_timestamps_sha11: text
- timeout_timestamps_sha11: text
- inactive_timestamps_sha11: text
- reward_timestamps_lga15: text

- reward_timestamps_lga16: text
- reward_timestamps_lga17: text
- reward_timestamps_lga18: text
- reward_timestamps_lga19: text
- reward_timestamps_lga20: text
- reward_timestamps_lga21: text
- reward_timestamps_lga22: text
- reward_timestamps_lga23: text
- reward_timestamps_lga24: text
- reward_timestamps_lga25: text
- reward_timestamps_lga26: text
- reward_timestamps_lga27: text
- reward_timestamps_lga28: text
- active_timestamps_lga15: text
- active_timestamps_lga16: text
- active_timestamps_lga17: text
- active_timestamps_lga18: text
- active_timestamps_lga19: text
- active_timestamps_lga20: text
- active_timestamps_lga21: text
- active_timestamps_lga22: text
- active_timestamps_lga23: text
- active_timestamps_lga24: text
- active_timestamps_lga25: text
- active_timestamps_lga26: text
- active_timestamps_lga27: text
- active_timestamps_lga28: text
- timeout_timestamps_lga15: text
- timeout_timestamps_lga16: text
- timeout_timestamps_lga17: text
- timeout_timestamps_lga18: text
- timeout_timestamps_lga19: text
- timeout_timestamps_lga20: text
- timeout_timestamps_lga21: text
- timeout_timestamps_lga22: text
- timeout_timestamps_lga23: text
- timeout_timestamps_lga24: text
- timeout_timestamps_lga25: text
- timeout_timestamps_lga26: text
- timeout_timestamps_lga27: text
- timeout_timestamps_lga28: text
- inactive_timestamps_lga15: text
- inactive_timestamps_lga16: text
- inactive_timestamps_lga17: text
- inactive_timestamps_lga18: text
- inactive_timestamps_lga19: text
- inactive_timestamps_lga20: text
- inactive_timestamps_lga21: text

- inactive_timestamps_lga22: text
- inactive_timestamps_lga23: text
- inactive_timestamps_lga24: text
- inactive_timestamps_lga25: text
- inactive_timestamps_lga26: text
- inactive_timestamps_lga27: text
- inactive_timestamps_lga28: text
- vf1_right_force_1: float
- vf1_right_force_2: float
- vf1_right_force_3: float
- vf1_right_force_avg: float
- vf1_right_time_1: float
- vf1_right_time_2: float
- vf1_right_time_3: float
- vf1_right_avg: float
- vf1_left_force_1: float
- vf1_left_force_2: float
- vf1_left_force_3: float
- vf1_left_force_avg: float
- vf1_left_time_1: float
- vf1_left_time_2: float
- vf1_left_time_3: float
- vf1_left_avg: float
- vf2_right_force_1: float
- vf2_right_force_2: float
- vf2_right_force_3: float
- vf2_right_force_avg: float
- vf2_right_time_1: float
- vf2_right_time_2: float
- vf2_right_time_3: float
- vf2_right_avg: float
- vf2_left_force_1: float
- vf2_left_force_2: float
- vf2_left_force_3: float
- vf2_left_force_avg: float
- vf2_left_time_1: float
- vf2_left_time_2: float
- vf2_left_time_3: float
- vf2_left_avg: float
- def_bsl_irr_1: NUMERIC
- agg_bsl_irr_1: NUMERIC
- irr_bsl_tot_1: NUMERIC
- def_bsl_irr_2: NUMERIC
- agg_bsl_irr_2: NUMERIC
- irr_bsl_total_2: NUMERIC
- def_bsl_irr_3: NUMERIC
- agg_bsl_irr_3: NUMERIC
- irr_bsl_total_3: NUMERIC

- def_drug_irr_1: NUMERIC
- agg_drug_irr_1: NUMERIC
- irr_drug_tot_1: NUMERIC
- def_drug_irr_2: NUMERIC
- agg_drug_irr_2: NUMERIC
- irr_drug_total_2: NUMERIC
- def_drug_irr_3: NUMERIC
- agg_drug_irr_3: NUMERIC
- irr_drug_total_3: NUMERIC
- measurement_value_feces_1: float
- measurement_value_feces_2: float
- measurement_value_feces_3: float
- measurement_value_feces_4: float
- measurement_value_urine_1: float
- measurement_value_urine_2: float
- measurement_value_weight_1: float
- measurement_value_weight_2: float
- measurement_value_weight_3: float
- measurement_value_weight_4: float
- measurement_value_weight_5: float
- measurement_value_weight_6: float
- measurement_value_weight_7: float
- measurement_value_weight_8: float
- measurement_value_weight_9: float
- measurement_value_weight_10: float
- age_at_arrival: integer
- age_at_surgery: integer
- age_at_lga: integer
- age_at_sha: integer
- age_at_dissection: integer
- date_of_birth: DATE
- date_of_death: DATE
- dissection_date: DATE
- short_access_start_date: DATE
- long_access_start_date: DATE
- von_frey_1_date: DATE
- von_frey_2_date: DATE
- shock_3_date: DATE
- recatheter_surgery_date: DATE
- progressive_ratio_1_date: DATE
- treatment_2_date: DATE
- tail_immersion_2_date: DATE
- female_swab_2_date: DATE
- treatment_4_date: DATE
- shock_1_date: DATE
- brevital_date: DATE
- tail_immersion_3_date: DATE
- lga_18_date: DATE

- long_access_end_date: DATE
- lga_17_date: DATE
- treatment_3_date: DATE
- lga_15_date: DATE
- pre_shock_date: DATE
- short_access_end_date: DATE
- irritability_1_date: DATE
- female_swab_1_date: DATE
- irritability_2_date: DATE
- surgeon: VARCHAR(255)
- recatheter_surgeon: VARCHAR(255)
- lga_19_date: DATE
- shock_2_date: DATE
- female_swab_3_date: DATE
- progressive_ratio_3_date: DATE
- handled_by: varchar(255)
- lga_pre_treatment_1_date: DATE
- lga_pre_treatment_3_date: DATE
- tail_immersion_1_date: DATE
- progressive_ratio_2_date: DATE
- lga_20_date: DATE
- surgery_assist: varchar(255)
- brevital_technicians: varchar(255)
- treatment_1_date: DATE
- lga_pre_treatment_2_date: DATE
- lga_pre_treatment_4_date: DATE
- lga_16_date: DATE
- lga_post_treatment_1_date: DATE
- lga_post_treatment_2_date: DATE
- lga_post_treatment_3_date: DATE
- lga_post_treatment_4_date: DATE
- exit_day: DATE
- date_of_wean: DATE
- date_of_ship: DATE
- arrival_date: DATE
- date_of_eye_bleed: DATE
- surgery_date: DATE
- irr_bsl_scorer_1: VARCHAR(255)
- irr_bsl_scorer_2: VARCHAR(255)
- irr_bsl_scorer_3: VARCHAR(255)
- irr_drug_scorer_1: VARCHAR(255)
- irr_drug_scorer_2: VARCHAR(255)
- irr_drug_scorer_3: VARCHAR(255)
- irritability_1_technicians: VARCHAR(255)
- irritability_2_technicians: VARCHAR(255)
- von_frey_1_technicians: VARCHAR(255)
- von_frey_2_technicians: VARCHAR(255)
- tail_immersion_1_technicians: VARCHAR(255)

- tail_immersion_2_technicians: VARCHAR(255)
- tail_immersion_3_technicians: VARCHAR(255)
- female_swab_1_technicians: VARCHAR(255)
- female_swab_2_technicians: VARCHAR(255)
- female_swab_3_technicians: VARCHAR(255)
- technician_urine_1: VARCHAR(255)
- technician_urine_2: VARCHAR(255)
- technician_feces_1: VARCHAR(255)
- technician_feces_2: VARCHAR(255)
- technician_feces_3: VARCHAR(255)
- technician_feces_4: VARCHAR(255)
- technician_weight_1: VARCHAR(255)
- technician_weight_2: VARCHAR(255)
- technician_weight_3: VARCHAR(255)
- technician_weight_4: VARCHAR(255)
- technician_weight_5: VARCHAR(255)
- technician_weight_6: VARCHAR(255)
- technician_weight_7: VARCHAR(255)
- technician_weight_8: VARCHAR(255)
- technician_weight_9: VARCHAR(255)
- technician_weight_10: VARCHAR(255)
- days_of_experiment: integer
- start_time_SHA01: TIME
- end_time_SHA01: TIME
- start_time_SHA02: TIME
- end_time_SHA02: TIME
- start_time_SHA03: TIME
- end_time_SHA03: TIME
- start_time_SHA04: TIME
- end_time_SHA04: TIME
- start_time_SHA05: TIME
- end_time_SHA05: TIME
- start_time_SHA06: TIME
- end_time_SHA06: TIME
- start_time_SHA07: TIME
- end_time_SHA07: TIME
- start_time_SHA08: TIME
- end_time_SHA08: TIME
- start_time_SHA09: TIME
- end_time_SHA09: TIME
- start_time_SHA10: TIME
- end_time_SHA10: TIME
- start_time_LGA01: TIME
- end_time_LGA01: TIME
- start_time_LGA02: TIME
- end_time_LGA02: TIME
- start_time_LGA03: TIME
- end_time_LGA03: TIME

- start_time_LGA04: TIME
- end_time_LGA04: TIME
- start_time_LGA05: TIME
- end_time_LGA05: TIME
- start_time_LGA06: TIME
- end_time_LGA06: TIME
- start_time_LGA07: TIME
- end_time_LGA07: TIME
- start_time_LGA08: TIME
- end_time_LGA08: TIME
- start_time_LGA09: TIME
- end_time_LGA09: TIME
- start_time_LGA10: TIME
- end_time_LGA10: TIME
- start_time_LGA11: TIME
- end_time_LGA11: TIME
- start_time_LGA12: TIME
- end_time_LGA12: TIME
- start_time_LGA13: TIME
- end_time_LGA13: TIME
- start_time_LGA14: TIME
- end_time_LGA14: TIME
- start_time_PR01: TIME
- end_time_PR01: TIME
- start_time_PR02: TIME
- end_time_PR02: TIME
- start_time_PR03: TIME
- end_time_PR03: TIME
- treatment_1_start_time: TIME
- treatment_2_start_time: TIME
- treatment_3_start_time: TIME
- treatment_4_start_time: TIME
- start_time_TREATMENT01: TIME
- end_time_TREATMENT01: TIME
- start_time_TREATMENT02: TIME
- end_time_TREATMENT02: TIME
- start_time_TREATMENT03: TIME
- end_time_TREATMENT03: TIME
- start_time_TREATMENT04: TIME
- end_time_TREATMENT04: TIME
- start_time_PRESHOCK: TIME
- end_time_PRESHOCK: TIME
- start_time_SHOCK_V1: TIME
- end_time_SHOCK_V1: TIME
- start_time_SHOCK_V2: TIME
- end_time_SHOCK_V2: TIME
- start_time_SHOCK_V3: TIME
- end_time_SHOCK_V3: TIME

- start_time_SHA11: TIME
- end_time_SHA11: TIME
- start_time_LGA15: TIME
- end_time_LGA15: TIME
- start_time_LGA16: TIME
- end_time_LGA16: TIME
- start_time_LGA17: TIME
- end_time_LGA17: TIME
- start_time_LGA18: TIME
- end_time_LGA18: TIME
- start_time_LGA19: TIME
- end_time_LGA19: TIME
- start_time_LGA20: TIME
- end_time_LGA20: TIME
- start_time_LGA21: TIME
- end_time_LGA21: TIME
- start_time_LGA22: TIME
- end_time_LGA22: TIME
- start_time_LGA23: TIME
- end_time_LGA23: TIME
- start_time_LGA24: TIME
- end_time_LGA24: TIME
- start_time_LGA25: TIME
- end_time_LGA25: TIME
- start_time_LGA26: TIME
- end_time_LGA26: TIME
- start_time_LGA27: TIME
- end_time_LGA27: TIME
- start_time_LGA28: TIME
- end_time_LGA28: TIME
- total_intake_lga integer
- total_intake_sha integer
- Shock_percent_rewards: float

Supplemental List 3. Variables in the stable combined SQL database are a selection of the most important variables in the raw database and derived dependent variables:

- **Selected important variables from the raw database**
  - rfid: varchar(255)
  - rfid: varchar
  - rat: varchar
  - cohort: int
  - sex: varchar
  - drug_group: varchar
  - experiment_group: varchar
  - dissection_group: varchar
  - brevital: varchar
  - last_good_session: varchar
  - exit_code: varchar
  - complete: varchar
  - tissue_collected: varchar
  - exit_notes: text
  - replaced_by: varchar
  - coat_color: varchar
  - ear_punch: varchar
  - litter_number: int
  - litter_size: int
  - rack: varchar
  - shipping_box: varchar
  - uv: varchar
  - female_swab_1_analysis: varchar
  - female_swab_2_analysis: varchar
  - female_swab_3_analysis: varchar
  - reward_presses_SHA01: int
  - reward_presses_SHA02: int
  - reward_presses_SHA03: int
  - reward_presses_SHA04: int
  - reward_presses_SHA05: int
  - reward_presses_SHA06: int
  - reward_presses_SHA07: int
  - reward_presses_SHA08: int
  - reward_presses_SHA09: int
  - reward_presses_SHA10: int
  - active_lever_presses_SHA01: int
  - active_lever_presses_SHA02: int
  - active_lever_presses_SHA03: int
  - active_lever_presses_SHA04: int
  - active_lever_presses_SHA05: int
  - active_lever_presses_SHA06: int
  - active_lever_presses_SHA07: int
  - active_lever_presses_SHA08: int
  - active_lever_presses_SHA09: int
  - active_lever_presses_SHA10: int

- timeout_presses_SHA01: int
- timeout_presses_SHA02: int
- timeout_presses_SHA03: int
- timeout_presses_SHA04: int
- timeout_presses_SHA05: int
- timeout_presses_SHA06: int
- timeout_presses_SHA07: int
- timeout_presses_SHA08: int
- timeout_presses_SHA09: int
- timeout_presses_SHA10: int
- inactive_lever_presses_SHA01: int
- inactive_lever_presses_SHA02: int
- inactive_lever_presses_SHA03: int
- inactive_lever_presses_SHA04: int
- inactive_lever_presses_SHA05: int
- inactive_lever_presses_SHA06: int
- inactive_lever_presses_SHA07: int
- inactive_lever_presses_SHA08: int
- inactive_lever_presses_SHA09: int
- inactive_lever_presses_SHA10: int
- reward_presses_LGA01: int
- reward_presses_LGA02: int
- reward_presses_LGA03: int
- reward_presses_LGA04: int
- reward_presses_LGA05: int
- reward_presses_LGA06: int
- reward_presses_LGA07: int
- reward_presses_LGA08: int
- reward_presses_LGA09: int
- reward_presses_LGA10: int
- reward_presses_LGA11: int
- reward_presses_LGA12: int
- reward_presses_LGA13: int
- reward_presses_LGA14: int
- active_lever_presses_LGA01: int
- active_lever_presses_LGA02: int
- active_lever_presses_LGA03: int
- active_lever_presses_LGA04: int
- active_lever_presses_LGA05: int
- active_lever_presses_LGA06: int
- active_lever_presses_LGA07: int
- active_lever_presses_LGA08: int
- active_lever_presses_LGA09: int
- active_lever_presses_LGA10: int
- active_lever_presses_LGA11: int
- active_lever_presses_LGA12: int
- active_lever_presses_LGA13: int
- active_lever_presses_LGA14: int

- timeout_presses_LGA01: int
- timeout_presses_LGA02: int
- timeout_presses_LGA03: int
- timeout_presses_LGA04: int
- timeout_presses_LGA05: int
- timeout_presses_LGA06: int
- timeout_presses_LGA07: int
- timeout_presses_LGA08: int
- timeout_presses_LGA09: int
- timeout_presses_LGA10: int
- timeout_presses_LGA11: int
- timeout_presses_LGA12: int
- timeout_presses_LGA13: int
- timeout_presses_LGA14: int
- inactive_lever_presses_LGA01: int
- inactive_lever_presses_LGA02: int
- inactive_lever_presses_LGA03: int
- inactive_lever_presses_LGA04: int
- inactive_lever_presses_LGA05: int
- inactive_lever_presses_LGA06: int
- inactive_lever_presses_LGA07: int
- inactive_lever_presses_LGA08: int
- inactive_lever_presses_LGA09: int
- inactive_lever_presses_LGA10: int
- inactive_lever_presses_LGA11: int
- inactive_lever_presses_LGA12: int
- inactive_lever_presses_LGA13: int
- inactive_lever_presses_LGA14: int
- reward_presses_PR01: int
- reward_presses_PR02: int
- reward_presses_PR03: int
- active_lever_presses_PR01: int
- active_lever_presses_PR02: int
- active_lever_presses_PR03: int
- inactive_lever_presses_PR01: int
- inactive_lever_presses_PR02: int
- inactive_lever_presses_PR03: int
- breakpoint_PR01: int
- breakpoint_PR02: int
- breakpoint_PR03: int
- last_ratio_PR01: int
- last_ratio_PR02: int
- last_ratio_PR03: int
- treatment_1_group: varchar
- treatment_2_group: varchar
- treatment_3_group: varchar
- treatment_4_group: varchar
- reward_presses_TREATMENT01: int

- reward_presses_TREATMENT02: int
- reward_presses_TREATMENT03: int
- reward_presses_TREATMENT04: int
- active_lever_presses_TREATMENT01: int
- active_lever_presses_TREATMENT02: int
- active_lever_presses_TREATMENT03: int
- active_lever_presses_TREATMENT04: int
- inactive_lever_presses_TREATMENT01: int
- inactive_lever_presses_TREATMENT02: int
- inactive_lever_presses_TREATMENT03: int
- inactive_lever_presses_TREATMENT04: int
- breakpoint_TREATMENT01: int
- breakpoint_TREATMENT02: int
- breakpoint_TREATMENT03: int
- breakpoint_TREATMENT04: int
- last_ratio_TREATMENT01: int
- last_ratio_TREATMENT02: int
- last_ratio_TREATMENT03: int
- last_ratio_TREATMENT04: int
- total_reward_PRESHOCK: int
- total_reward_SHOCK_V1: int
- total_reward_SHOCK_V2: int
- total_reward_SHOCK_V3: int
- total_active_lever_presses_PRESHOCK: int
- total_active_lever_presses_SHOCK_V1: int
- total_active_lever_presses_SHOCK_V2: int
- total_active_lever_presses_SHOCK_V3: int
- total_inactive_lever_presses_PRESHOCK: int
- total_inactive_lever_presses_SHOCK_V1: int
- total_inactive_lever_presses_SHOCK_V2: int
- total_inactive_lever_presses_SHOCK_V3: int
- total_shocks_PRESHOCK: int
- total_shocks_SHOCK_V1: int
- total_shocks_SHOCK_V2: int
- total_shocks_SHOCK_V3: int
- rewards_after_first_shock_PRESHOCK: text
- rewards_after_first_shock_SHOCK_V1: text
- rewards_after_first_shock_SHOCK_V2: text
- rewards_after_first_shock_SHOCK_V3: text
- reward_presses_SHA11: int
- active_lever_presses_SHA11: int
- timeout_presses_SHA11: int
- inactive_lever_presses_SHA11: int
- reward_presses_LGA15: int
- reward_presses_LGA16: int
- reward_presses_LGA17: int
- reward_presses_LGA18: int
- reward_presses_LGA19: int

- reward_presses_LGA20: int
- reward_presses_LGA21: int
- reward_presses_LGA22: int
- reward_presses_LGA23: int
- reward_presses_LGA24: int
- reward_presses_LGA25: int
- reward_presses_LGA26: int
- reward_presses_LGA27: int
- reward_presses_LGA28: int
- active_lever_presses_LGA15: int
- active_lever_presses_LGA16: int
- active_lever_presses_LGA17: int
- active_lever_presses_LGA18: int
- active_lever_presses_LGA19: int
- active_lever_presses_LGA20: int
- active_lever_presses_LGA21: int
- active_lever_presses_LGA22: int
- active_lever_presses_LGA23: int
- active_lever_presses_LGA24: int
- active_lever_presses_LGA25: int
- active_lever_presses_LGA26: int
- active_lever_presses_LGA27: int
- active_lever_presses_LGA28: int
- timeout_presses_LGA15: int
- timeout_presses_LGA16: int
- timeout_presses_LGA17: int
- timeout_presses_LGA18: int
- timeout_presses_LGA19: int
- timeout_presses_LGA20: int
- timeout_presses_LGA21: int
- timeout_presses_LGA22: int
- timeout_presses_LGA23: int
- timeout_presses_LGA24: int
- timeout_presses_LGA25: int
- timeout_presses_LGA26: int
- timeout_presses_LGA27: int
- timeout_presses_LGA28: int
- inactive_lever_presses_LGA15: int
- inactive_lever_presses_LGA16: int
- inactive_lever_presses_LGA17: int
- inactive_lever_presses_LGA18: int
- inactive_lever_presses_LGA19: int
- inactive_lever_presses_LGA20: int
- inactive_lever_presses_LGA21: int
- inactive_lever_presses_LGA22: int
- inactive_lever_presses_LGA23: int
- inactive_lever_presses_LGA24: int
- inactive_lever_presses_LGA25: int

- inactive_lever_presses_LGA26: int
- inactive_lever_presses_LGA27: int
- inactive_lever_presses_LGA28: int
- von_frey_1_force: float
- von_frey_1_time: float
- von_frey_2_force: float
- von_frey_2_time: float
- von_frey_difference_force: float
- tail_immersion_1_time: float
- tail_immersion_2_time: float
- tail_immersion_3_time: float
- tail_immersion_difference_tolerance: float
- def_bsl_ave: numeric
- agg_bsl_ave: numeric
- total_bsl_ave: numeric
- def_drug_ave: numeric
- agg_drug_ave: numeric
- total_drug_ave: numeric
- diff_ave_def: numeric
- diff_ave_agg: numeric
- diff_ave_total: numeric
- reward_timestamps_SHA01: text
- reward_timestamps_SHA02: text
- reward_timestamps_SHA03: text
- reward_timestamps_SHA04: text
- reward_timestamps_SHA05: text
- reward_timestamps_SHA06: text
- reward_timestamps_SHA07: text
- reward_timestamps_SHA08: text
- reward_timestamps_SHA09: text
- reward_timestamps_SHA10: text
- active_timestamps_SHA01: text
- active_timestamps_SHA02: text
- active_timestamps_SHA03: text
- active_timestamps_SHA04: text
- active_timestamps_SHA05: text
- active_timestamps_SHA06: text
- active_timestamps_SHA07: text
- active_timestamps_SHA08: text
- active_timestamps_SHA09: text
- active_timestamps_SHA10: text
- timeout_timestamps_SHA01: text
- timeout_timestamps_SHA02: text
- timeout_timestamps_SHA03: text
- timeout_timestamps_SHA04: text
- timeout_timestamps_SHA05: text
- timeout_timestamps_SHA06: text
- timeout_timestamps_SHA07: text

- timeout_timestamps_SHA08: text
- timeout_timestamps_SHA09: text
- timeout_timestamps_SHA10: text
- inactive_timestamps_SHA01: text
- inactive_timestamps_SHA02: text
- inactive_timestamps_SHA03: text
- inactive_timestamps_SHA04: text
- inactive_timestamps_SHA05: text
- inactive_timestamps_SHA06: text
- inactive_timestamps_SHA07: text
- inactive_timestamps_SHA08: text
- inactive_timestamps_SHA09: text
- inactive_timestamps_SHA10: text
- reward_timestamps_LGA01: text
- reward_timestamps_LGA02: text
- reward_timestamps_LGA03: text
- reward_timestamps_LGA04: text
- reward_timestamps_LGA05: text
- reward_timestamps_LGA06: text
- reward_timestamps_LGA07: text
- reward_timestamps_LGA08: text
- reward_timestamps_LGA09: text
- reward_timestamps_LGA10: text
- reward_timestamps_LGA11: text
- reward_timestamps_LGA12: text
- reward_timestamps_LGA13: text
- reward_timestamps_LGA14: text
- active_timestamps_LGA01: text
- active_timestamps_LGA02: text
- active_timestamps_LGA03: text
- active_timestamps_LGA04: text
- active_timestamps_LGA05: text
- active_timestamps_LGA06: text
- active_timestamps_LGA07: text
- active_timestamps_LGA08: text
- active_timestamps_LGA09: text
- active_timestamps_LGA10: text
- active_timestamps_LGA11: text
- active_timestamps_LGA12: text
- active_timestamps_LGA13: text
- active_timestamps_LGA14: text
- timeout_timestamps_LGA01: text
- timeout_timestamps_LGA02: text
- timeout_timestamps_LGA03: text
- timeout_timestamps_LGA04: text
- timeout_timestamps_LGA05: text
- timeout_timestamps_LGA06: text
- timeout_timestamps_LGA07: text

- timeout_timestamps_LGA08: text
- timeout_timestamps_LGA09: text
- timeout_timestamps_LGA10: text
- timeout_timestamps_LGA11: text
- timeout_timestamps_LGA12: text
- timeout_timestamps_LGA13: text
- timeout_timestamps_LGA14: text
- inactive_timestamps_LGA01: text
- inactive_timestamps_LGA02: text
- inactive_timestamps_LGA03: text
- inactive_timestamps_LGA04: text
- inactive_timestamps_LGA05: text
- inactive_timestamps_LGA06: text
- inactive_timestamps_LGA07: text
- inactive_timestamps_LGA08: text
- inactive_timestamps_LGA09: text
- inactive_timestamps_LGA10: text
- inactive_timestamps_LGA11: text
- inactive_timestamps_LGA12: text
- inactive_timestamps_LGA13: text
- inactive_timestamps_LGA14: text
- ratios_PR01: text
- ratios_PR02: text
- ratios_PR03: text
- ratios_TREATMENT01: text
- ratios_TREATMENT02: text
- ratios_TREATMENT03: text
- ratios_TREATMENT04: text
- group_pre_shock: int
- group_shock: int
- rewards_got_shock_PRESHOCK: text
- rewards_got_shock_SHOCK_V1: text
- rewards_got_shock_SHOCK_V2: text
- rewards_got_shock_SHOCK_V3: text
- reward_timestamps_PRESHOCK: text
- reward_timestamps_SHOCK_V1: text
- reward_timestamps_SHOCK_V2: text
- reward_timestamps_SHOCK_V3: text
- reward_timestamps_SHA11: text
- active_timestamps_SHA11: text
- timeout_timestamps_SHA11: text
- inactive_timestamps_SHA11: text
- reward_timestamps_LGA15: text
- reward_timestamps_LGA16: text
- reward_timestamps_LGA17: text
- reward_timestamps_LGA18: text
- reward_timestamps_LGA19: text
- reward_timestamps_LGA20: text

- reward_timestamps_LGA21: text
- reward_timestamps_LGA22: text
- reward_timestamps_LGA23: text
- reward_timestamps_LGA24: text
- reward_timestamps_LGA25: text
- reward_timestamps_LGA26: text
- reward_timestamps_LGA27: text
- reward_timestamps_LGA28: text
- active_timestamps_LGA15: text
- active_timestamps_LGA16: text
- active_timestamps_LGA17: text
- active_timestamps_LGA18: text
- active_timestamps_LGA19: text
- active_timestamps_LGA20: text
- active_timestamps_LGA21: text
- active_timestamps_LGA22: text
- active_timestamps_LGA23: text
- active_timestamps_LGA24: text
- active_timestamps_LGA25: text
- active_timestamps_LGA26: text
- active_timestamps_LGA27: text
- active_timestamps_LGA28: text
- timeout_timestamps_LGA15: text
- timeout_timestamps_LGA16: text
- timeout_timestamps_LGA17: text
- timeout_timestamps_LGA18: text
- timeout_timestamps_LGA19: text
- timeout_timestamps_LGA20: text
- timeout_timestamps_LGA21: text
- timeout_timestamps_LGA22: text
- timeout_timestamps_LGA23: text
- timeout_timestamps_LGA24: text
- timeout_timestamps_LGA25: text
- timeout_timestamps_LGA26: text
- timeout_timestamps_LGA27: text
- timeout_timestamps_LGA28: text
- inactive_timestamps_LGA15: text
- inactive_timestamps_LGA16: text
- inactive_timestamps_LGA17: text
- inactive_timestamps_LGA18: text
- inactive_timestamps_LGA19: text
- inactive_timestamps_LGA20: text
- inactive_timestamps_LGA21: text
- inactive_timestamps_LGA22: text
- inactive_timestamps_LGA23: text
- inactive_timestamps_LGA24: text
- inactive_timestamps_LGA25: text
- inactive_timestamps_LGA26: text

- inactive_timestamps_LGA27: text
- inactive_timestamps_LGA28: text
- vf1_right_force_1: float
- vf1_right_force_2: float
- vf1_right_force_3: float
- vf1_right_force_avg: float
- vf1_right_time_1: float
- vf1_right_time_2: float
- vf1_right_time_3: float
- vf1_right_avg: float
- vf1_left_force_1: float
- vf1_left_force_2: float
- vf1_left_force_3: float
- vf1_left_force_avg: float
- vf1_left_time_1: float
- vf1_left_time_2: float
- vf1_left_time_3: float
- vf1_left_avg: float
- vf2_right_force_1: float
- vf2_right_force_2: float
- vf2_right_force_3: float
- vf2_right_force_avg: float
- vf2_right_time_1: float
- vf2_right_time_2: float
- vf2_right_time_3: float
- vf2_right_avg: float
- vf2_left_force_1: float
- vf2_left_force_2: float
- vf2_left_force_3: float
- vf2_left_force_avg: float
- vf2_left_time_1: float
- vf2_left_time_2: float
- vf2_left_time_3: float
- vf2_left_avg: float
- def_bsl_irr_1: numeric
- agg_bsl_irr_1: numeric
- irr_bsl_tot_1: numeric
- def_bsl_irr_2: numeric
- agg_bsl_irr_2: numeric
- irr_bsl_total_2: numeric
- def_bsl_irr_3: numeric
- agg_bsl_irr_3: numeric
- irr_bsl_total_3: numeric
- def_drug_irr_1: numeric
- agg_drug_irr_1: numeric
- irr_drug_tot_1: numeric
- def_drug_irr_2: numeric
- agg_drug_irr_2: numeric

- irr_drug_total_2: numeric
- def_drug_irr_3: numeric
- agg_drug_irr_3: numeric
- irr_drug_total_3: numeric
- measurement_value_feces_1: float
- measurement_value_feces_2: float
- measurement_value_feces_3: float
- measurement_value_feces_4: float
- measurement_value_urine_1: float
- measurement_value_urine_2: float
- measurement_value_weight_1: float
- measurement_value_weight_2: float
- measurement_value_weight_3: float
- measurement_value_weight_4: float
- measurement_value_weight_5: float
- measurement_value_weight_6: float
- measurement_value_weight_7: float
- measurement_value_weight_8: float
- measurement_value_weight_9: float
- measurement_value_weight_10: float
- age_at_arrival: int
- age_at_surgery: int
- age_at_lga: int
- age_at_sha: int
- age_at_dissection: int
- esc_aio: float
- pr_aio: float
- withdrawal_aio: float
- tolerance_aio: float
- ai_aio: float
- lga_mean_delta_esc_11_14: float
- shock_aio: float
- sha_mean_delta_esc_08_10: float
- esc_i_s: float
- pr_i_s: float
- withdrawal_i_s: float
- tolerance_i_s: float
- ai_s: float
- shock_i_s: float
- esc_i_sc: float
- pr_i_sc: float
- withdrawal_i_sc: float
- tolerance_i_sc: float
- ai_sc: float
- shock_i_sc: float
- lga_iti_median_01_02: float
- lga_iti_coefficient_of_variation_01_02: float
- lga_iti_mode_01_02: float

- lga_iti_median_11_14: float
- lga_iti_coefficient_of_variation_11_14: float
- lga_iti_mode_11_14: float
- lga_mean_01_02: float
- lga_mean_11_14: float
- lga_mean_active_01_02: float
- lga_mean_active_11_14: float
- lga_mean_inactive_01_02: float
- lga_mean_inactive_11_14: float
- lga_mean_to_01_02: float
- lga_mean_to_11_14: float
- sha_iti_median_03_04: float
- sha_iti_coefficient_of_variation_01_02: float
- sha_iti_coefficient_of_variation_03_04: float
- sha_iti_mode_01_02: float
- sha_iti_mode_03_04: float
- sha_mean_01_02: float
- sha_mean_03_04: float
- sha_mean_inactive_01_02: float
- sha_mean_inactive_03_04: float
- sha_mean_to_01_02: float
- sha_mean_to_03_04: float
- sha_mean_01_03: float
- sha_mean_08_10: float
- sha_mean_inactive_01_03: float
- sha_mean_inactive_08_10: float
- sha_mean_to_01_03: float
- sha_mean_to_08_10: float
- pr_vehicle_active: float
- pr_vehicle_breakpoint: float
- pr_vehicle_inactive: float
- pr_vehicle_rewards: float
- treatments_pr_vehicle: float
- pr_saline_active: float
- pr_saline_breakpoint: float
- pr_saline_inactive: float
- pr_saline_rewards: float
- treatments_pr_saline: float
- pr_methadone_active: float
- pr_methadone_breakpoint: float
- pr_methadone_inactive: float
- pr_methadone_rewards: float
- treatments_pr_methadone: float
- pr_naltrexone_active: float
- pr_naltrexone_breakpoint: float
- pr_naltrexone_inactive: float
- pr_naltrexone_rewards: float
- treatments_pr_naltrexone: float

- lga_loading_phase_intake_01_02: float
- lga_titration_phase_01_02: float
- lga_loading_phase_intake_11_14: float
- lga_titration_phase_11_14: float
- sha_iti_numburst_01_02: float
- sha_iti_numburst_03_04: float
- sha_iti_maxburst_01_02: float
- sha_iti_maxburst_03_04: float
- sha_iti_meannumrewards_01_02: float
- sha_iti_meannumrewards_03_04: float
- sha_loading_phase_intake_01_02: float
- sha_titration_phase_01_02: float
- sha_loading_phase_intake_03_04: float
- sha_titration_phase_03_04: float
- sha_loading_phase_intake_01_03: float
- sha_titration_phase_01_03: float
- sha_loading_phase_intake_08_10: float
- sha_titration_phase_08_10: float
- lga_iti_numburst_01_02: float
- lga_iti_numburst_11_14: float
- lga_iti_maxburst_01_02: float
- lga_iti_maxburst_11_14: float
- lga_iti_meannumrewards_01_02: float
- lga_iti_meannumrewards_11_14: float
- lga_iti_pctnumrewards_01_02: float
- lga_iti_pctnumrewards_11_14: float
- tail_immersion_analgesia: float
- tail_immersion_tolerance: float
- tail_immersion_baseline: float
- tail_immersion_oxypresa: float
- tail_immersion_oxypostsa: float
- pr_01_sha: float
- pr_01_inactive: float
- pr_01_active: float
- pr_01_sha_breakpoint: float
- pr_02_lga: float
- pr_02_inactive: float
- pr_02_active: float
- pr_02_lga_breakpoint: float
- pr_03_postshock: float
- pr_03_postshock_breakpoint: float
- pr_max_02_03: float
- pr_max_02_03_breakpoint: float
- von_frey_force_bsl: float
- von_frey_force_difference: float
- von_frey_force_percent: float
- von_frey_force_withdrawl: float
- von_frey_time_bsl: float

- von_frey_time_difference: float
- von_frey_time_percent: float
- von_frey_time_withdrawl: float
- sha_behavior_stability_01_03: float
- sha_coefficient_of_variation_2: float
- sha_behavior_stability_08_10: float
- sha_coefficient_of_variation: float
- sha_iti_median_08_10: float
- sha_behavior_stability_08_10_na: float
- lga_behavior_stability_11_14: float
- lga_behavior_stability_11_14_na: float
- shock_03: float
- shock_percentage_pre: float
- shock_03_pre: float
- irr_agg_change: float
- irr_def_change: float
- irr_total_change: float
- surgery_date: date
- date_of_birth: date
- date_of_death: date
- dissection_date: date
- short_access_start_date: date
- long_access_start_date: date
- von_frey_1_date: date
- von_frey_2_date: date
- shock_3_date: date
- total_intake_lga: int
- total_intake_sha: int
- shock_percent_rewards: float
- ai_aio_quartile: int
- ai_s_quartile: int
- ai_sc_quartile: int
- ai_aio_quartile_str: text
- ai_s_quartile_str: text
- ai_sc_quartile_str: text
- esc_sha: float
- esc_sha_i_s: float
- esc_sha_i_sc: float
- intake_ai_aio_quartile: float
- intake_ai_s_quartile: float
- intake_ai_sc_quartile: float
- intake_ai_aio_quartile_str: text
- intake_ai_s_quartile_str: text
- intake_ai_sc_quartile_str: text
- intake_ai_aio: float
- intake_ai_s: float
- intake_ai_sc: float

- **New calculated dependent variable**
  - Add_ind_olivier: Average of z-scores for escalation (Esc), motivation (PR), tolerance and pain subset by cohort and sex. Esc = z-score of average of escalation indices for LgA 12, 13, and 14, Esc index xx = (rewards LgAxx - average rewards LgA01)/ st.dev. LgA01, PR = z-score of number of rewards obtained during PR2 (LgA), Tolerance = z-score of (tail immersion 2 - tail immersion 3), Pain = z-score of (Force BSL - Force withdrawal)"
  - Add_index_sexcohort_z: index calculated by Palmer team
  - Addiction_index_no_sex_z: index calculated by Palmer team with no seperate Zscore of male and female
  - Irr_agg_change: aggressive behavior counts after self admin - aggressive behavior counts before self admin
  - Irr_def_change: defensive behavior counts after self admin - defensive behavior counts before self admin
  - Irr_total_change: aggressive and defensive behavior counts after self admin - aggressive and defensive behavior counts before self admin
  - Lga_behavior_stability_11_14: standard deviation of median (no NA correction) intertrial interval in LGA (days 11-14)
  - Lga_behavior_stability_11_14_na: standard deviation of median (correcting NA's with 0's if NA's come from trials with one/two rewards) intertrial interval in LGA (days 11-14)
  - Lga_coefficient_of_variation_11_14: standard deviation of ITI/mean of ITI within LGA (days 11-14)
  - Lga_iti_coefficient_of_variation_01_02: standard deviation of ITI/mean of ITI for first 2 LGA days
  - Lga_iti_maxburst_01_02: maximum number of rewards contain in a single burst in one session for first 2 LGA days
  - Lga_iti_maxburst_12_14: maximum number of rewards contain in a single burst in one session for last 4 LGA days
  - Lga_iti_meannumrewards_01_02: average number of rewards in a burst over all bursts in the session for first 2 LGA days
  - Lga_iti_meannumrewards_11_14: average number of rewards in a burst over all bursts in the session for last 4 LGA days
  - lga_iti_median_01_02: median of ITI (ITI being the time in sec between reward events using the timestamp data) for first 2 LGA days
  - Lga_iti_median_11_14: Median of ITI (ITI being the time in sec between reward events using the timestamp data)
  - Lga_iti_mode_01_02: most frequent ITI (ITI being the time in sec between reward events using the timestamp data) for first 2 LGA days
  - Lga_iti_mode_11_14: most frequent ITI (ITI being the time in sec between reward events using the timestamp data) for last 4 LGA days
  - Lga_iti_numburst_01_02: number of bursts (= grouped rewards that are separated by max 120 sec) in a session for first 2 LGA days
  - Lga_iti_numburst_11_14: number of bursts (= grouped rewards that are separated by max 120 sec) in a session for last 4 LGA days
  - Lga_iti_pctrewards_01_02: percantage of rewards that fall in a burst of total rewards for the session for first 2 LGA days
  - Lga_iti_pctrewards_11_14: percantage of rewards that fall in a burst of total rewards for the session for last 4 LGA days

- Lga_loading_phase_intake_01_02: number of rewards during the first ten minutes of first 2 LGA days
- Lga_loading_phase_intake_11_14: number of rewards during the first ten minutes of LGA (days 11 -14)
- Lga_mean_01_02: average of the number of oxycodone infusions during the first 2 LGA days
- Lga_mean_11_14: average of the number of cocaine infusions during the last three sessions of long access self administration (6 hours)
- Lga_mean_active_01_02: average of the active during the first 2 LGA days
- Lga_mean_active_11_14: average of the active during the last 4 LGA days
- Lga_mean_delta_esc: average of the escalation values during the last 3 LGA days
- Lga_mean_delta_esc_11_14: average of the escalation values during the last three sessions of long access self administration (6 hours)
- Lga_mean_inactive_01_02: average of the inactive during the first 2 LGA days
- Lga_mean_inactive_11_14: average of the inactive during the last three sessions of long access self administration (6 hours)
- Lga_mean_to_01_02: average of the TO escalation values during the first 2 LGA days (# active - # rewards (because not every active lever press leads to reward))
- Lga_mean_to_11_14: average of the TO escalation values during the last 4 LGA days (# active - # rewards (because not every active lever press leads to reward))
- Lga_titration_phase_01_02: number of rewards during the last 6 * 60 min in first 2 LGA days (hours 5-11)
- Lga_titration_phase_11_14: number of rewards during the last 6 * 60 min in last 4 LGA days (hours 5-11)
- Pr_01_active: number of active lever presses during day 1 (post-ShA) of progressive ratio session
- pr_01_inactive: number of inactive lever presses during day 1 (post-ShA) of progressive ratio session
- Pr_01_sha: number of cocaine infusions received during day 1 (post-ShA) of progressive ratio at breakpoint
- Pr_01_sha_breakpoint: last completed number of oxycodone active lever presses during day 1 (post-ShA) of progressive ratio at breakpoint
- Pr_02_active: number of active lever presses during day 2 (post-LgA) of progressive ratio session
- Pr_02_inactive: number of inactive lever presses during day 2 (post-LgA) of progressive ratio session
- Pr_02_lga: number of cocaine infusions received during day 2 (post-LgA) of progressive ratio at breakpoint
- Pr_02_lga: number of oxycodone infusions received during day 2 (post-LgA)
- pr_02_lga_breakpoint: last completed number of oxycodone active lever presses during day 2 (post-ShA) of progressive ratio at breakpoint
- Pr_03_postshock: number of cocaine infusions received during day 3 (post shock) of progressive ratio at breakpoint
- Pr_03_postshock_breakpoint: number of cocaine infusions received during day 3 (post shock) of progressive ratio at breakpoint
- Pr_buprenorphine_active: number of active lever presses during buprenorphine treatment
- Pr_buprenorphine_breakpoint: breakpoint (last completed ratio) during buprenorphine treatment
- Pr_buprenorphine_inactive: number of inactive lever presses during buprenorphine treatment

- Pr_buprenorphine_rewards: number of oxycodone infusions received during buprenorphine treatment
- Pr_max_02_03: max value between pr2&3
- Pr_max_02_03_breakpoint: max value between pr2&3
- Pr_methadone_active: number of active lever presses during methadone treatment
- Pr_methadone_breakpoint: breakpoint (last completed ratio) during methadone treatment
- Pr_methadone_inactive: number of inactive lever presses during methadone treatment
- Pr_methadone_rewards: number of oxycodone infusions received during methadone treatment
- Pr_naltrexone_active: number of active lever presses during naltrexone treatment
- Pr_naltrexone_breakpoint: breakpoint (last completed ratio) during naltrexone treatment
- Pr_naltrexone_inactive: number of inactive lever presses during naltrexone treatment
- Pr_naltrexone_rewards: number of oxycodone infusions received during naltrexone treatment
- Pr_saline_active: number of active lever presses during saline treatment
- pr_saline_breakpoint breakpoint (last completed ratio) during saline treatment
- Pr_saline_inactive: number of inactive lever presses during saline treatment
- Pr_saline_rewards: number of oxycodone infusions received during saline treatment
- Sha_behavior_stability_01_03: standard deviation of median (no NA correction) intertrial interval in SHA (days 01-03)
- Sha_behavior_stability_08_10: standard deviation of median (no NA correction) intertrial interval in SHA (days 08-10)
- Sha_behavior_stability_08_10_na: standard deviation of median (correcting NA's with 0's if NA's come from trials with one/two rewards) intertrial interval in SHA (days 08-10)
- Sha_coefficient_of_variation: standard deviation of ITI/mean of ITI within SHA (days 08-10)
- Sha_coefficient_of_variation_2: standard deviation of ITI/mean of ITI within SHA (days 01-03)
- Sha_iti_coefficient_of_variation_01_02: standard deviation of ITI/mean of ITI for first 2 SHA days
- Sha_iti_coefficient_of_variation_03_04: standard deviation of ITI/mean of ITI for last 2 SHA days
- Sha_iti_maxburst_01_02: maximum number of rewards contain in a single burst in one session for first 2 SHA days
- Sha_iti_maxburst_03_04: maximum number of rewards contain in a single burst in one session for last 2 SHA days
- Sha_iti_meannumrewards_01_02: average number of rewards in a burst over all bursts in the session for first 2 SHA days
- Sha_iti_meannumrewards_03_04: average number of rewards in a burst over all bursts in the session for last 2 SHA days
- Sha_iti_median_01_02: median of ITI (ITI being the time in sec between reward events using the timestamp data) for first 2 SHA days
- Sha_iti_median_03_04: median of ITI (ITI being the time in sec between reward events using the timestamp data) for last 2 SHA days
- Sha_iti_median_08_10: Median of ITI (ITI being the time in sec between reward events using the timestamp data)
- Sha_iti_mode_01_02: most frequent ITI (ITI being the time in sec between reward events using the timestamp data) for first 2 SHA days
- Sha_iti_mode_03_04: most frequent ITI (ITI being the time in sec between reward events using the timestamp data) for last 2 SHA days
- Sha_iti_numburst_01_02: number of bursts (= grouped rewards that are seperated by max 120 sec) in a session for first 2 SHA days

- Sha_iti_numburst_03_04: number of bursts (= grouped rewards that are seperated by max 120 sec) in a session for last 2 SHA days
- Sha_iti_pctrewards_01_02: percantage of rewards that fall in a burst of total rewards for the session for first 2 SHA days
- Sha_iti_pctrewards_03_04: percantage of rewards that fall in a burst of total rewards for the session for last 2 SHA days
- Sha_loading_phase_intake_01_02: number of rewards during the first ten minutes of first 2 SHA days
- Sha_loading_phase_intake_01_03: number of rewards during the first ten minutes of SHA (days 01-03)
- Sha_loading_phase_intake_03_04: number of rewards during the first ten minutes of last 2 SHA days
- Sha_loading_phase_intake_08_10: number of rewards during the first ten minutes of SHA (days 08-10)
- Sha_mean_01_02: average of the number of oxycodone infusions during the first 2 SHA days
- Sha_mean_01_03: average of the number of cocaine infusions during the first three sessions of short access self administration (2 hours)
- Sha_mean_03_04: average of the number of oxycodone infusions during the last 2 SHA days
- Sha_mean_08_10: average of the number of cocaine infusions during the last three sessions of short access self administration (2 hours)
- Sha_mean_active_01_02: average of the active during the first 2 SHA days
- Sha_mean_active_03_04: average of the active during the last 2 SHA days
- Sha_mean_delta_esc_08_10: average of the escalation values during the last three sessions of short access self administration (2 hours)
- Sha_mean_inactive_01_02: average of the inactive during the first 2 SHA days
- Sha_mean_inactive_01_03: average of the number of inactive during the first three sessions of short access self administration (2 hours)
- Sha_mean_inactive_03_04: average of the inactive during the last 2 SHA days
- Sha_mean_inactive_08_10: average of the number of inactive during the last three sessions of short access self administration (2 hours)
- Sha_mean_to_01_02: average of the TO escalation values during the first 2 SHA days (# active - # rewards (because not every active lever press leads to reward))
- Sha_mean_to_01_03: average of the number of TO response during the first three sessions of short access self administration (2 hours)
- Sha_mean_to_03_04: average of the TO escalation values during the last 2 SHA days (# active - # rewards (because not every active lever press leads to reward))
- Sha_mean_to_08_10: average of the number of TO response during the last three sessions of short access self administration (2 hours)
- Sha_titration_phase_01_02: number of rewards during the last 60 min in first 2 SHA days (hours 1-2)
- Sha_titration_phase_01_03: intake during the last 60 min in SHA (days 01-03)
- Sha_titration_phase_03_04: number of rewards during the last 60 min in last 2 SHA days (hours 1-2)
- Sha_titration_phase_08_10: intake during the last 60 min in SHA (days 08-10)
- Shock_03: number of cocaine infusions received during foot shocks of 0.3 mA (For all shock data: omit rewards received before the first shock; Total rewards - (M:1:) +1 =rewards after first shock > includes the reward that accompanies the first shock)

- Shock_percentage_Pre: number of rewards after first shock = A, B = total rewards in preshock/total rewards in first hour of last 3 days: A/B *100
- Shock_03_pre: % shock_0.3 vs preshock session (For all shock data: omit rewards received before the first shock; Total rewards - (M:1:) +1 =rewards after first shock > includes the reward that accompanies the first shock)
- Tail_immersion_analgesia: change in latency (tail imm 2 - tail imm BSL) note: positive number is more analgesia
- Tail_immersion_baseline: baseline latency (tail imm BSL)
- Tail_immersion_oxypostsa: tail imm 3
- Tail_immersion_oxypresa: tail imm 2
- Tail_immersion_tolerance: change in latency (tail imm 2 - tail imm 3) note: positive number is more tolerance
- Treatments_pr_buprenorphine: percent of vehicle for each pharmacological treatment ((treatment PR - PR02) / PR02 x 100%) (buprenorphine)
- Treatments_pr_methadone: percent of vehicle for each pharmacological treatment ((treatment PR - PR02) / PR02 x 100%) (methadone)
- Treatments_pr_naltrexone: percent of vehicle for each pharmacological treatment ((ttreatment PR - PR02) / PR02 x 100%) (naltrexone)
- Treatments_pr_vehicle: percent of vehicle for each pharmacological treatment ((treatment PR - PR02) / PR02 x 100%) (vehicle)
- Von_frey_force_bsl: threshold force at baseline
- Von_frey_force_difference: change in average force achieved (BSL - Withdrawal)
- Von_frey_force_percent: percent change in average force achieved (BSL-Withdrawal) / BSL*100
- Von_frey_force_withdrawal: threshold force in withdrawal
- Von_frey_time_bsl: threshold time at baseline
- Von_frey_time_difference: change in average time achieved (BSL - Withdrawal)
- Von_frey_time_percent: percent change in average time achieved (BSL-Withdrawal) / BSL*100
- Von_frey_time_withdrawal: threshold time in withdrawal
